# Multiple Eurasian Origins and Admixture Shaped the Genomic Legacy of the House Mouse Invasion in Africa

**DOI:** 10.1111/mec.70507

**Published:** 2026-08-13

**Authors:** Daniel Poveda‐Martinez, Philippe Gauthier, Margaux J. M. Lefebvre, Fanny Degrugillier, Gauthier Dobigny, Jean‐Pierre Quéré, Ambroise Dalecky, Youssoupha Niang, Mamadou Kane, Clark Mbou‐Boutambe, Joa Mangombi, Solimane Ag Atteynine, Sylvestre Badou, Madougou Garba, Pierre Caminade, Barthélémy Ngoubangoye, Carole M. Smadja, Larson Boundenga, Laurent Granjon, Virginie Rougeron, Franck Prugnolle, Carine Brouat

**Affiliations:** ^1^ CBGP, IRD, CIRAD, INRAE, Institut Agro, University of Montpellier Montpellier France; ^2^ Department of Archaeogenetics Max Planck Institute for Evolutionary Anthropology Leipzig Germany; ^3^ Laboratoire MIVEGEC University of Montpellier, CNRS‐UM, IRD Montpellier France; ^4^ Unité Peste Institut Pasteur de Madagascar Antananarivo Madagascar; ^5^ BIOPASS 2, IRD, Cirad, ISRA, Université Gaston Berger Saint‐Louis Senegal; ^6^ LPED IRD, Aix Marseille Université Marseille France; ^7^ Centre Interdisciplinaires de Recherches Médicales de Franceville (CIRMF) Franceville Gabon; ^8^ Institut d'Economie Rurale (IER) Sotuba Mali; ^9^ Ecole Polytechnique d'Abomey Calavi (EPAC) Université d'Abomey Calavi (UAC) d'Abomey‐Calavi Benin; ^10^ Direction Générale de la Protection des Végétaux (DGPV) Niamey Niger; ^11^ Institut des Sciences de l'Evolution de Montpellier (ISEM) CNRS, IRD, University of Montpellier Montpellier France; ^12^ REHABS, International Research Laboratory, CNRS‐NMU, George Campus Nelson Mandela University George South Africa; ^13^ Sustainability Research Unit, George Campus Nelson Mandela University George South Africa

**Keywords:** Africa, biological invasions, commensal rodents, introgression, population genomics

## Abstract

Commensal mammals offer unique opportunities to study how human‐mediated dispersal, admixture, and secondary contact shape genomic variation during range expansion, yet for one of the most successful invasive commensals globally, the western house mouse *Mus musculus domesticus* , invasion dynamics across Africa remain largely unexplored. Here, we present a large‐scale population genomic analysis of 380 whole‐genome sequences, including 303 newly sequenced low‐coverage genomes, of which 216 are from 13 distinct African populations, enabling us to investigate how human‐mediated dispersal and secondary contact with native species shape the genomic legacy of a commensal mammal invasion. Ancestry analyses reveal contributions from at least three major Eurasian source lineages contributing to African populations: an Iberian–West Asian lineage present in Morocco, Algeria, and Niger; a Mediterranean lineage represented in Tunisia; and a Northern European lineage dominant in West and Central Africa. Demographic inferences indicate both recent and ancient episodes of divergence and population contraction. In West and Central Africa, divergence times broadly overlap with European maritime expansion and colonial trade. In contrast, North African populations exhibit older coalescent signals, consistent with early participation in the western Mediterranean radiation. We also detect substantial introgression from the native *Mus spretus* into North African populations in regions of sympatry. Collectively, our results reveal that the African invasion of the house mouse was not a single demographic process but a mosaic of population‐specific histories shaped by multiple introductions, shifting connectivity through time, admixture, and interspecific gene flow. These findings reinforce the importance of population‐level perspectives in invasion genomics, especially for commensal mammals whose dispersal is closely tied to human movement, and establish the most extensive open genomic resource for wild African house mice.

## 1 Introduction

Human‐mediated biological invasions provide powerful natural experiments for understanding how demographic processes shape genomic variation during range expansion. Founder effects, multiple introductions, secondary contact zones, introgression from resident taxa, and shifting connectivity through time all leave detectable genomic signatures, while occurring within historically tractable windows (Estoup and Guillemaud 2010; Excoffier et al. 2009). Among invasive organisms, commensal species are especially informative because their dispersal is not merely correlated with human expansion, but often directly driven by it through trade networks, maritime routes, agricultural frontiers, and urbanisation (Hulme‐Beaman et al. 2016). These dispersal vectors are independently documented through archaeological, zooarchaeological, and historical records (Cucchi et al. 2005, 2020), providing explicit prior hypotheses about the timing, directionality, and frequency of introduction events that are rarely available for non‐commensal invasive species. Commensal invasions can therefore serve as models for understanding how introductions, admixture among divergent source populations, and secondary contact with native congeners shape the evolutionary trajectory of invading lineages, and for testing whether genomic signals of invasion history align with the human movement, trade, and settlement networks that drove them.

One of the most well‐documented examples is the western house mouse, *Mus musculus domesticus* , whose commensal relationship with humans is believed to have originated around 15 thousand years ago (kya) in Western Asia (Cucchi et al. 2020). Thereafter, this subspecies, part of the *Mus musculus* species complex, spread alongside human populations across Europe, the Americas, Australasia, and Africa, following waves of global exploration, settlement and trade (Phifer‐Rixey and Nachman 2015; Jones et al. 2013; Macholán et al. 2012; Suzuki et al. 2013). Today, alongside commensal rats such as the brown rat ( *Rattus norvegicus* ) and black rat ( *Rattus rattus* ), the western house mouse is recognised as one of the world's most successful invasive mammal species, owing to its global distribution and strong ecological plasticity (Munshi‐South et al. 2024; Capizzi et al. 2014; Phifer‐Rixey and Nachman 2015).

In recent years, population genetic studies have elucidated the evolutionary origin of *M. m. domesticus* (Hardouin et al. 2015; Harr et al. 2016; Phifer‐Rixey et al. 2020; Phifer‐Rixey et al. 2018; Suzuki et al. 2013; Chen et al. 2025) and traced its spread and colonisation of diverse regions of the world over the last several millennia (Jones et al. 2013; Gray et al. 2014; Gabriel et al. 2015; Morgan et al. 2022; Babiker and Tautz 2015; Veale et al. 2018; Hardouin et al. 2010). These genetic insights align closely with zooarchaeological evidence (Cucchi et al. 2005, 2020) and even parallel patterns observed in human population genomics, revealing a striking concordance between mouse and human population genomic variation (Agwamba et al. 2025). Altogether, research on wild house mice highlights their longstanding commensal relationship with humans and the central role of human migration in shaping the genomic landscape of this key model species.

In particular, genetic signatures of human‐mediated range expansion have been extensively characterised in wild house mouse populations across Europe (Harr et al. 2016; Morgan et al. 2022; Agwamba et al. 2025), offshore islands (Hardouin et al. 2010; Morgan et al. 2022), and North America (Agwamba and Nachman 2023), substantially advancing our understanding of European mouse population structure and extra‐European dispersal patterns. In Europe, three major genetic clusters have been identified, Mediterranean, Northern European, and Atlantic Iberian, with their diversification dating to approximately 1.5 to 5.5 kya (Agwamba et al. 2025). Outside Europe, genomic data have revealed multiple, relatively recent colonisation events in North American house mouse populations, primarily originating from distinct Western European sources (Agwamba and Nachman 2023). Western European ancestry predominates in these populations, although the precise European origins remain uncertain and may involve contributions from both British and Spanish lineages (Agwamba and Nachman 2023). Similarly, house mouse populations on various oceanic islands exhibit a predominance of Northern European ancestry (Hardouin et al. 2010; Morgan et al. 2022), illustrating the central role of European colonial expansion in shaping the global distribution of *M. m. domesticus*.

In contrast, the invasion dynamics of the western house mouse within Africa remained largely unexplored, although this continent has played a pivotal role in shaping recent human history through migration and trade, particularly since the colonial period. The Atlantic slave trade was one of the largest maritime migrations in history, and involved the large‐scale massive transoceanic movement of people and goods, linking Europe, Africa, and the Americas (Klein 2010; Eltis and Richardson 2008). Today, Africa remains deeply integrated into global exchange networks, underscoring its central role in both ancient and recent processes of human‐mediated dispersal. Yet, the genetic legacy of these movements in commensal species such as *M. m. domesticus* remains surprisingly understudied. To date, our understanding of house mouse colonisation in Africa has relied on a few genetic studies that together provide only a fragmented picture of its introduction and dispersal across the continent (Hardouin et al. 2010; Bonhomme et al. 2011; Suzuki et al. 2013; Lippens et al. 2017). The first large‐scale study, by Bonhomme et al. (2011), analysed mitochondrial sequences from six populations across Egypt, Tunisia, Morocco, Algeria, Senegal, and Cameroon, suggesting that *M. m. domesticus* colonised North Africa from the Near East during Antiquity, whereas West Africa was colonised from Western Europe during the colonial period. This latter scenario was further supported by Lippens et al. (2017), who combined mitochondrial and microsatellite markers in Senegal and inferred an initial introduction along the northern Atlantic coast followed by a southward expansion, consistent with European colonial settlement and trade routes.

Range expansion through human‐mediated dispersal repeatedly brings invasive lineages into contact with both closely related taxa and native species, creating opportunities for hybridisation and interspecific gene flow that can profoundly shape the genomic variation of invading populations. In *M. m. domesticus*, such contacts have been documented in multiple contexts (Phifer‐Rixey and Nachman 2015; Liu et al. 2015; Banker et al. 2022; Orth et al. 1998; McCormick et al. 2014). Secondary contact with the sister subspecies *M. m. musculus* occurs across a well‐characterised hybrid zone in Europe (Phifer‐Rixey and Nachman 2015), while admixture with *M. m. castaneus* has been documented in invasive populations in North America and New Zealand, in some cases contributing non‐trivial ancestry to introduced populations (Orth et al. 1998; McCormick et al. 2014). Beyond intraspecific contacts, genomic studies in the western Mediterranean have revealed substantial interspecific gene flow between *M. m. domesticus* and the Algerian mouse, *Mus spretus* , a native species distributed across North Africa and parts of southwestern Europe (Liu et al. 2015; Banker et al. 2022). Despite strong postzygotic reproductive barriers (Matsuda and Chapman 1992), introgression from *M. spretus* into *M. m. domesticus* has been described, with gene flow decreasing with distance from European zones of sympatry (Banker et al. 2022). As *M. m. domesticus* has expanded into North Africa, where *M. spretus* is native, the extent of hybridisation in African sympatric zones, and whether contact with *M. spretus* has left confounding genomic signatures in the invading population, remain open questions with direct implications for interpreting the colonisation history of the house mouse on the continent.

Here, we present a large‐scale population genomic analysis of 380 whole‐genome sequences representing *M. m. domesticus* populations across West Asia, Europe, North America and critically a broad geographic range in North and sub‐saharan Africa. This dataset includes 303 newly generated low‐coverage genomes, 216 of which derive from 13 African populations, providing the most comprehensive genomic resource to date for wild *M. m. domesticus* in the continent. Specifically, we ask when and from where the western house mouse colonised the African continent. We hypothesise multiple temporally distinct introduction events, an early arrival into North Africa during Antiquity (3000–400 bce), and a more recent colonisation of coastal West Africa during the European maritime expansion (15th–19th centuries), followed by inland dispersal facilitated by human transportation networks. By integrating these new genomic data with previously published genome sequences of the native mouse *M. spretus* , we then test for asymmetric allele sharing consistent with introgression in North African regions of sympatry and evaluate whether similar signals occur in allopatry in West and Central Africa. Finally, we quantify how invasion history has shaped genetic diversity by comparing heterozygosity and genomic inbreeding among African populations. Together, our analyses provide new insights into the invasion dynamics of the house mouse in Africa, and update models of house mouse spread within Europe and North America. By incorporating a dense genomic sampling across Africa, our study fills a major gap in the global invasion history of *M. m. domesticus* and establishes the largest open genomic resource for wild African house mice. More broadly, we provide a missing piece of the puzzle, positioning Africa as a crucial region for understanding how human‐mediated dispersal, admixture, and secondary contact shape the demographic and genomic legacy of commensal mammal invasions.

## 2 Methods

### 2.1 Sample Collection and Genome Sequencing

**FIGURE 1:**
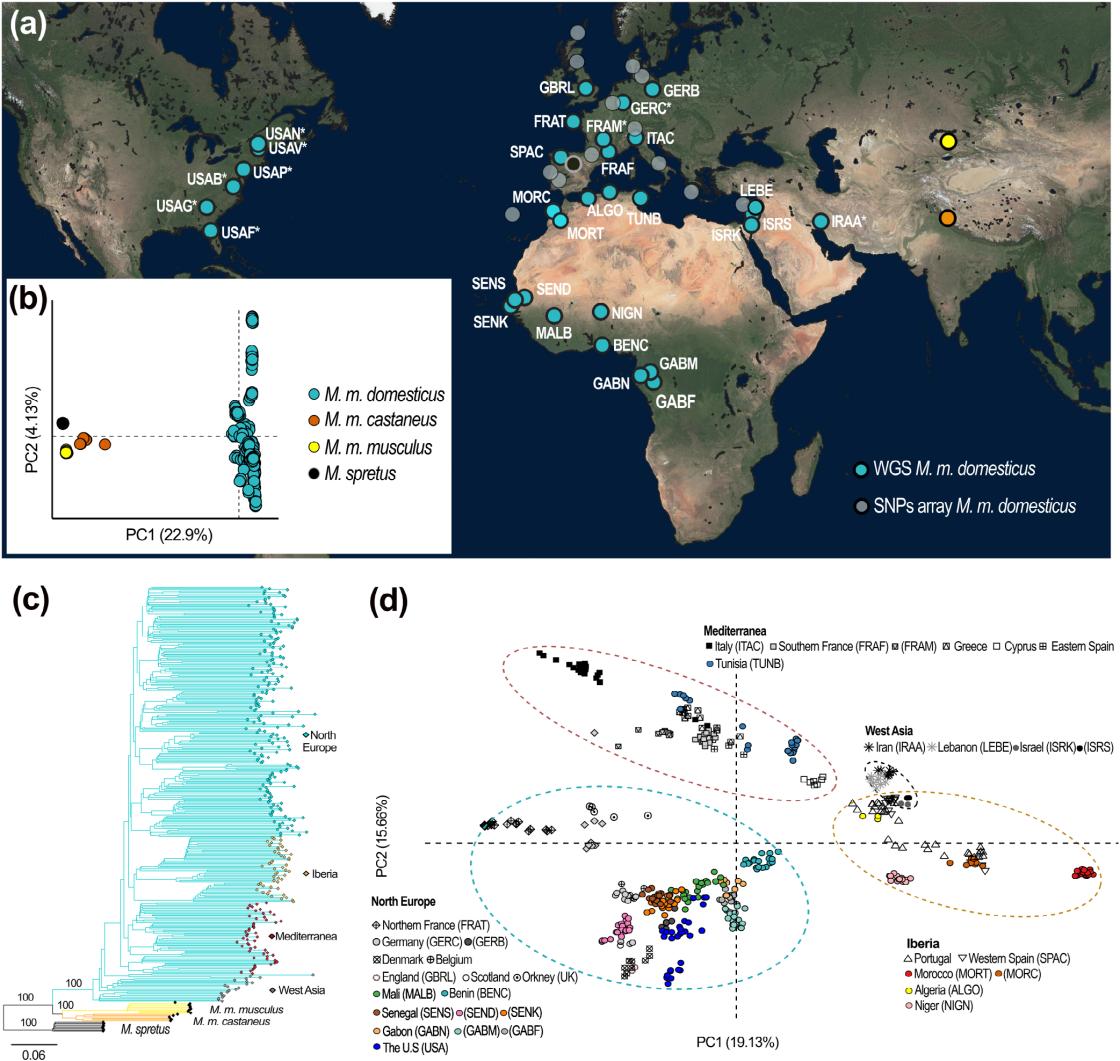
Sampling, genetic structure and origin of the African house mouse populations. (a) Geographic sampling of 380 house mouse genomes, showing population codes and species included in this study. The panel also includes the distribution of *M. m. domesticus* populations previously genotyped using SNP arrays (Morgan et al. 2022) that were incorporated in this analysis. An asterisk (*) next to *M. m. domesticus* populations codes indicate genomes obtained from previously published studies (Harr et al. 2016; Phifer‐Rixey et al. 2018). (b) Principal component analysis (PCA) of a multispecies dataset (340 unrelated genomes) including *M. m. domesticus*, *M. m. castaneus, M. m. musculus*, and *M. spretus* , showing genetic clustering by taxon. (c) Genotype‐likelihood–based phylogeny of the *Mus musculus* species complex. *M. m. domesticus* forms a single, strongly supported clade that encompasses the Northern European, Mediterranean, and Iberian populations together with Western Asian populations. *M. m. musculus* and *M. m. castaneus* each form well‐supported lineages. (d) PCA of a merged *M. m. domesticus* dataset (471 house mice) using PLINK, revealing three major genetic clusters in which African populations fall: Northern Europe, Mediterranean, and Iberian. The Mediterranean cluster includes populations from southern Europe as well as Tunisia in North Africa. The Iberian cluster comprises populations from the Iberian Peninsula together with populations from Morocco and Algeria in North Africa, as well as Niger. The Northern European cluster encompasses African populations from West and Central Africa, as well as all North American populations. Four‐letter population codes, as shown in panel a, are used for the whole‐genome sequenced samples, while the full country name is displayed for the SNP‐array‐genotyped populations included in this dataset.

This study generated 303 new whole‐genome sequences of *M. m. domesticus* samples from eight African countries (13 populations; 216 samples), five European countries (6 populations, 73 samples), and three countries in the species' native range in West Asia (3 populations, 14 samples) (Figure 1a). Only the samples from Gabon (Ndjolé) were collected specifically for this study during fieldwork conducted in 2022, whereas all other specimens were obtained from existing scientific and museum collections, with collection dates spanning 1984 to 2022 (Table S1). Sampling was conducted in urban or peri‐urban localities; notably, the Cotonou (Benin) population was collected within a port infrastructure, whereas other sampling sites were not directly associated with major ports. Whole‐genome sequencing was performed to all samples at low coverage (lcWGS) (2.4× mean depth, individual sample depths are provided in Table S1) using 150 bp paired‐end reads mode on the Illumina NovaSeq 6000 platform (Illumina Inc., San Diego, CA, USA). Additional sampling and laboratory details are provided in Table S1 and Note S1.

To complement our newly generated dataset, we retrieved publicly available whole‐genome sequencing (WGS) data, including 24 *M. m. domesticus* samples from Europe and West Asia (PRJEB9450; average depth of ∼21×) (Harr et al. 2016), and 30 from the United States (PRJNA397406; ∼5×) (Phifer‐Rixey et al. 2018); seven *M. m. castaneus* samples from India (PRJEB2176; ∼17×) (Harr et al. 2016); eight *M. m. musculus* from Kazakhstan (PRJEB11742; ∼24×) (Harr et al. 2016); and eight *M. spretus* from Spain (PRJEB11742; ∼22×) (Harr et al. 2016). To further expand geographic coverage, we incorporated genotype data from 114 *M. m. domesticus* individuals from Europe, generated using exome sequencing of approximately 50,000 SNPs (Morgan et al. 2022).

### 2.2 Data Processing, Mapping and Genotype Calling

Raw sequencing reads were processed using fastp v0.23.2 and trimmed reads were mapped to the chromosome‐level assemblies against the house mouse GRCm39 reference genome using BWA‐MEM v0.7.18. Reads were post‐processed using SAMtools v1.20, and putative PCR duplicates were flagged using the ‘markdup’ command of Sambamba v1.0.1. Resulting BAM files were filtered to remove unmapped reads, PCR duplicates, and read pairs that mapped to different contigs outside the 19 autosomal chromosomes. For genotype calling of single‐nucleotide polymorphism (SNP), we used the probabilistic framework based on genotype likelihoods implemented in ANGSD v.0.940 (Korneliussen et al. 2014), as an alternative to calling genotypes to process both lcWGS and WGS samples. We use the Snakemake pipeline, loco‐pipe (low‐coverage pipeline) (Zhou et al. 2024), to automate the ANGSD workflow. Loco‐pipe incorporates the best practices and filtering steps for lcWGS data (Lou et al. 2021; Zhou et al. 2024). Genotype likelihoods were estimated using the GATK model implemented in ANGSD (‐GL 2). Missing data in these datasets were handled through site‐level sample‐representation and read‐depth filters. Specifically, during global SNP discovery, we required at least 50% of individuals in a given dataset to have a read depth of at least one read at a site (minind_proportion = 0.5). Sites represented in fewer than half of the individuals were therefore excluded. We further retained only sites passing standard lcWGS read‐level filters, including minimum mapping quality 30, minimum base quality 30, removal of bad reads, uniquely mapping reads, and properly paired reads (Lou et al. 2021). SNPs were retained using a minimum minor allele frequency of 0.05 and a SNP discovery threshold of *p* < 1 × 10^−6^. We assessed relatedness and removed close relatives and duplicates using ngsRelate v2 (Hanghøj et al. 2019). Pairs with KING‐robust kinship > 0.177 (first‐degree relatives) were filtered by retaining one individual per pair, resulting in the removal of 40 samples. The final dataset comprised 340 unrelated genomes spanning *M. musculus* subspecies and *M. spretus* (multispecies dataset) and 317 unrelated *M. m. domesticus* genomes (western house mouse dataset), each with an average of approximately 10 individuals per population (Table S1). Additional details on ANGSD genotype‐likelihood estimation and relatedness filtering are provided in Note S2.

### 2.3 Genotype Imputation

We performed genotype imputation on 357 genomes, including both low‐coverage and high coverage *M. m. domesticus* genomes. Imputation was necessary because several downstream analyses and software tools used in this study, such as Relate (Speidel et al. 2019), require called genotypes rather than genotype likelihoods, which are inherently uncertain at low coverage. Imputation addresses this issue by statistically inferring the most likely genotypes at each site, leveraging linkage disequilibrium patterns and shared haplotypes across individuals (Teng et al. 2022). Genotypes were imputed with STITCH (Davies et al. 2016) using only sites passing initial QC and detected as variable in ANGSD (SNP *p*‐value < 1 × 10^−6^). After imputation, the dataset contained 19,998,106 SNPs. To retain high‐confidence imputed sites and limit missing data, we filtered SNPs using an imputation information score ≥ 0.9 and retained only sites genotyped in at least 90% of individuals. This produced a final imputed house mouse dataset of 14,981,900 SNPs. Individual‐level missingness in this filtered dataset ranged from 0.55% to 13.27% across individuals, with a mean of 2.54%. Imputation accuracy was assessed using 19 high‐coverage *M. m. domesticus* genomes from three European populations (Harr et al. 2016; mean depth ~21×) as a validation set, comparing imputed genotypes against these high‐confidence calls. Additional details on STITCH settings, construction of the validation dataset, and accuracy results are provided in Note S3.

### 2.4 Merging WGS Dataset and SNPs Array Genotypes

To further expand the geographic coverage in our dataset, we merged the imputed dataset with exome genotype dataset from 114 additional unrelated *M. m. domesticus* individuals from Europe (Morgan et al. 2022) (Table S4). Coordinates of the exome‐derived SNPs were lifted over to the GRCm39/mm39 genome assembly using the UCSC chain file mm10ToMm39.over.chain.gz, and overlapping sites between the SNP‐array and imputed WGS datasets were identified using bcftools *isec* v1.9. The initial filtered imputed WGS dataset contained 14,981,900 SNPs, whereas the exome dataset contained 57,945 sites. After retaining only shared biallelic SNPs between both datasets, the merged dataset contained 11,247 SNPs across 471 individuals, representing 0.075% of the imputed WGS SNPs and 19.41% of the exome‐derived sites. Individual‐level missingness in the merged dataset ranged from 0.18% to 12.27%, with a mean of 1.91%. The full list of datasets generated in this study, the samples included in each, and the corresponding downstream analyses in which each dataset was used are detailed Tables S1, S2 and Figure S1.

### 2.5 Species Assignment and Population Structure

We explored species assignment and population structure on the multispecies and western house mouse datasets using different population genetic methods. For each dataset, principal component analysis (PCA) was executed using the genotype likelihoods in Beagle format generated in ANGSD, with additional filters to remove tri‐allelic sites, SNPs with a minor allele frequency of 0.05, keep only unlinked SNPs, and require each retained site to be represented by at least 50% of individuals. We used PCAngsd v0.99 (Meisner and Albrechtsen 2018) to estimate the covariance matrix and identify potentially species clustering in the multispecies dataset. In parallel, we inferred a genotype‐likelihood–based phylogeny to further validate species assignments. Pairwise genetic distances were computed from genotype likelihoods (ANGSD Beagle format) using ngsDist v1.0.10 (Vieira et al. 2016) with 100 bootstrap replicates (block size = 1000 sites). Phylogenetic trees were reconstructed with FastME v2.1.6.1 (Lefort et al. 2015). In addition to the PCAs based on genotype likelihoods, we conducted a PCA using PLINK v2 (Purcell et al. 2007) on genotyped data from the merged house mouse dataset which includes both imputed whole‐genome and exome‐genotyped samples (471 samples and 11,274 sites; mean individual‐level missingness = 1.91%) to expand the coverage and increase the population structure resolution within our study area.

Admixture proportions for the western house mouse dataset were inferred using two complementary approaches. First, NGSadmix (Skotte et al. 2013), based on the same Beagle genotype likelihoods file used in PCAngsd, was ran for *K* = 2 to *K* = 9 until the model converged, where the top three maximum likelihood runs were within 10 log‐likelihood units of each other or until a limit of 4000 independent runs was reached without convergence. Since NGSadmix only works with genotype likelihoods, we used in addition the sparse non‐negative matrix factorisation approach (sNMF) (Frichot et al. 2014), as implemented in LEA R package (Frichot and François 2015) to infer the admixture proportion on individuals from the merged house mouse dataset. Such coefficients were calculated for *K* values ranging from 2 to 9, with 1000 replicates per *K* and 9999 iterations per run.

### 2.6 Population Genetic Relationships and Introgression

Relationships between populations in the western house mouse dataset, while taking into account gene flow between populations, were estimated using two complementary approaches, TreeMix v1.13 (Pickrell and Pritchard 2012) and AdmixtureBayes (Nielsen et al. 2023). Only populations with more than five individuals in the western house mouse dataset were included in analyses (ISRK, ISRS, GBRL, and ALGO were excluded). We used the eight *M. spretus* genomes as an outgroup. The optimal number of migration events (*m*) was determined by running 50 replicates for each *m* value, ranging from 0 to 10. The optimal number of migration events was identified using the OptM R package, which identifies the best‐fitting model based on the second derivative of the log‐likelihood, and the results were further post‐processed with the BITE R package. For AdmixtureBayes analysis, three independent runs were performed, each consisting of 40 Markov chain models and 250,000 steps to ensure convergence after discarding the first 50% as burn‐in. The number of retained admixture events was evaluated based on their posterior probabilities.

To investigate signatures of introgression between African house mouse populations and the native *M. spretus* , we performed *D*‐statistics (Durand et al. 2011) using the doAbbababa2 module in ANGSD (Korneliussen et al. 2014). This approach allowed us to test for excess allele sharing while accounting for genotype uncertainty in low‐coverage sequencing data. The analysis was conducted for the triplets of populations P1, P2 and P3. As P1 we used the more distant population of *M. m. domesticus* (IRAA, from Iran); as P2 we used the house mouse populations from Africa, Europe, or North America, and as P3 we used *M. spretus* populations. *Mus caroli* sequences were included as an outgroup (Thybert et al. 2018). We applied the ‐doAbbaBaba2 in ANGSD across the entire genome, using all available variable sites, to maximise statistical power in detecting excess allele sharing between P2 and P3 or P1 and P3 populations. A block jackknife procedure was applied to estimate standard errors and calculate *Z*‐scores. To complement *D*‐statistics and quantify the relative magnitude of introgression, we also estimated the admixture proportion *f* (Martin et al. 2015). This statistic scales the observed excess of ABBA over BABA sites relative to the expectation under complete admixture, providing a genome‐wide estimate of the proportion of ancestry shared through introgression (Martin et al. 2015).

### 2.7 Demographic Inference of the House Mouse in Africa

To investigate the demographic history and invasion dynamics of the house mouse in Africa, we used Relate (Speidel et al. 2019), a genealogy‐based approach that estimates genome‐wide genealogies as trees, accounting for local ancestry changes due to recombination. We applied Relate to 357 individuals from the imputed house mouse dataset, analysing approximately 14 million SNPs with a mean individual‐level missingness of 2.54%. Haplotype phasing required by Relate was conducted using Shapeit v2.0 (Delaneau et al. 2019). We performed this analysis using an effective population size of 106,000 individuals, as previously estimated for *M. m. domesticus* (Phifer‐Rixey et al. 2020), a mutation rate of 4.1 × 10^−9^ per site per generation (Geraldes et al. 2011). This mutation rate was selected to ensure comparability with recent demographic studies based on whole‐genome data for *M. m. domesticus* (Chen et al. 2025; Agwamba et al. 2025), while remaining within the empirically supported range for the species (Geraldes et al. 2011; Milholland et al. 2017; Phifer‐Rixey et al. 2020). For recombination, we used the genetic map estimated from contemporary patterns of linkage disequilibrium for a wild European house mouse population (Wooldridge and Dumont 2023).

To characterise population separation histories among major *M. m. domesticus* lineages and between European, West Asia, African and North American populations, we applied MSMC‐IM (Wang et al. 2020), which fits an isolation‐with‐migration (IM) model to time‐resolved coalescence rates. The pairs of populations used here correspond to well‐defined genetic clusters in population structure and phylogenetic analyses. For each comparison, we first extracted the sub‐genealogy corresponding to the individuals of interest from the full Relate genealogies and re‐estimated within‐group and cross‐group coalescence rates following Speidel et al. (2019) using the *FinalizePopulationSize* module from Relate. Conversion of Relate output files into the MSMC‐IM input format was performed using custom scripts kindly provided by E. Patin and D. Liu (Institut Pasteur, Paris). MSMC‐IM was then run for each population pair to estimate (i) the cross‐coalescence rate (CCR) through time and (ii) the corresponding population split time (defined as the point where CCR = 0.5, following Wang et al. 2020) which corresponds to the time at which lineages are equally likely to coalesce within either population, indicating the midpoint of divergence between the two populations, rather than a strict bifurcation event. All MSMC‐IM analyses assumed a mutation rate of *μ* = 4.1 × 10^−9^ per site per generation. To account for uncertainty in generation time, divergence times were interpreted under two alternative scaling assumptions: two generations per year (0.5 years per generation) (Bronson 1979) and one generation per year (Geraldes et al. 2008). Effective population sizes required by MSMC‐IM were taken from the population‐size trajectories estimated by Relate.

To detect founder effects and invasion‐associated bottlenecks, we inferred historical changes in effective population size (*Nₑ*) using two complementary approaches: Relate, which estimates *Nₑ* from genome‐wide genealogies, and Stairway Plot 2 (Liu and Fu 2020), which reconstructs *Nₑ* trajectories from the site‐frequency spectrum (SFS) and is sensitive to recent contractions. With relate, we estimated the population sizes for the 28 populations in the imputed house mouse dataset. To assess the robustness of these population size inferences to parameter assumptions, we additionally performed sensitivity analyses using alternative mutation rates (*μ* = 4.1 × 10^−9^ and *μ* = 5.7 × 10^−9^ per site per generation). For Stairway Plot 2, we estimated a genome‐wide SFS, and restricted our analysis to populations with sample sizes > 10 individuals. Populations were intentionally merged prior to SFS estimation to improve SFS stability. This approach was applied to Morocco and Algeria, Senegal, Gabon, and North America populations, which were grouped based on their respective ancestry profiles inferred from population structure analyses, as well as TreeMix and AdmixtureBayes results. The SFS was estimated using the easySFS.py script (https://github.com/isaacovercast/easySFS) applied to pre‐filtered VCF files. We used a stringent imputation quality threshold of ≥ 0.99 and a missing‐data filter of ≤ 1%, which together provide a reasonable compromise for low‐coverage WGS while discarding low‐confidence imputed genotypes.

### 2.8 Genomic Diversity and Inbreeding Inference From HBD Segments

To investigate the consequences of colonisation bottlenecks on genetic diversity and inbreeding, we analysed genome‐wide patterns across populations in the western house mouse dataset using genotype‐likelihood–based estimators in ANGSD. Individual heterozygosity was derived from single‐individual SAF files. We estimated each individual SFS with realSFS (Nielsen et al. 2012) and computed heterozygosity as the proportion of heterozygous sites over the total number of analysed sites. We also estimated population‐level diversity (nucleotide diversity *π* and Watterson's *θ*), and Tajima's D, across autosomes by first generating population SAF files in ANGSD (−doSaf) and then using realSFS to infer *π* and *θ* with SAF‐based priors. Diversity and neutrality statistics were estimated in non‐overlapping 250 kb windows using a 250 kb window size and 250 kb step size. Genome‐wide *π* and *θ* were obtained by summing autosome‐specific estimates and dividing by the total number of analysed sites. Tajima's *D* was summarised at the autosome level for each population and plotted as the distribution of autosome‐specific values. To infer genomic inbreeding in African house mouse populations, we used RZooRoH v0.4.1 (Druet and Gautier 2017; Bertrand et al. 2019), which detects homozygosity‐by‐descent (HBD) segments from genotype likelihoods using a hidden Markov model (HMM), explicitly accounting for genotype uncertainty at low coverage. We fitted an HMM comprising 14 HBD classes plus a non‐HBD class, with class rates following an exponential series (Rk = 2*ⁿ*, *n* = 1…14), enabling partitioning of autozygosity into age‐related components (shorter tracts reflecting older inbreeding). For each individual, we estimated the genomic inbreeding coefficient *F*_HBD_ as the realised fraction of the autosomal genome assigned to any HBD class (mean posterior HBD probability across markers). We summarised the contribution of each HBD class to describe the age structure of inbreeding, and tested differences in *F*_HBD_ among populations/regions using linear models.

## 3 Results

To investigate the origins and invasion dynamics of the house mouse in Africa, we sequenced 303 low‐coverage whole genomes (~2.4× mean coverage) of *M. m. domesticus*, including 216 from 13 African populations, 73 from seven European populations, and 14 from two West Asian populations (Figure 1a and Table S1). We added 54 published *M. m. domesticus* genomes from Europe (*n* = 16), West Asia (8), and North America (30), as well as 23 genomes from *M. m. castaneus* (7), *M. m. musculus* (8), and *M. spretus* (8), totaling 380 genomes. After removing 40 first‐degree relatives where necessary, we assembled four datasets: (i) a multispecies dataset of 340 unrelated genomes (23.1 million SNPs; 23,110 unlinked SNPs); (ii) a western house mouse dataset of 317 unrelated *M. m. domesticus* genomes (19.5 million SNPs; 19,542 unlinked SNPs); (iii) an imputed *M. m. domesticus* dataset comprising 357 genomes with 14.9 million SNPs, generated by imputing genotype calls from low‐coverage sequencing data; and (iv) a merged dataset of 471 individuals combining 114 SNP‐array‐genotyped mice (Morgan et al. 2022) with overlapping whole‐genome data (11,247 SNPs). To minimise uncertainty from low sequencing depth, downstream analyses were performed using genotype likelihoods whenever possible; hard genotype calls were restricted to analyses that require called or phased genotypes (see Methods, Table S2 and Figure S1).

### 3.1 Population Structure and Origins of the African House Mouse Populations

We examined broad‐scale population structure using multivariate and phylogenetic analyses of the multispecies dataset to contextualise African house mouse populations within the diversity of the *Mus musculus* species complex. PCA revealed four genetic clusters broadly corresponding to the nominal taxonomic assignments of the sampled mice (Figure 1b). In the multispecies PCA, *M. m. domesticus* individuals were clearly separated from *M. m. musculus, M. m. castaneus*, and *M. spretus* . Phylogenetic reconstruction was consistent with the PCA patterns (Figures 1c and S2). All *M. m. domesticus* samples grouped into a single, well‐supported clade that included Northern European, Mediterranean, Iberian, and Western Asian populations. Outside this assemblage, *M. m. musculus* and *M. m. castaneus* formed separate clades, while *M. spretus* was recovered as the most divergent lineage.

We next examined genetic relationships between African *M. m. domesticus* populations and those from other regions using two datasets: the whole‐genome western house mouse dataset, offering high‐resolution markers, and the merged dataset, which provides broader geographic coverage, particularly of potential European source populations. PCAs based on both datasets revealed consistent patterns of genetic structure, highlighting genetic affinities between African populations and those from Europe and West Asia (Figures 1d and S3). We hereafter describe the results obtained using the merged dataset, which includes a broader geographic sampling (Figure 1d). This analysis revealed three main genetic clusters. The Mediterranean cluster spans southern Europe (Cyprus, Greece, Italy, southern France, and eastern Spain) and includes Tunisia (TUNB). The Iberian cluster comprises populations from Portugal and western Spain, as well as North African populations from Algeria (ALGO), Morocco (MORC, MORT), and Niger (NIGN). West Asian populations from Iran, Lebanon, and Israel cluster closely with the Iberian and Mediterranean clusters. The Northern Europe cluster includes populations from northern France, Switzerland, Germany, Denmark, Belgium, and the United Kingdom, together with African populations from Senegal (SENS, SEND, SENK), Mali (MALB), Gabon (GABF, GABM, GABN), and Benin (BENC). This cluster also encompasses all North American populations.

**FIGURE 2:**
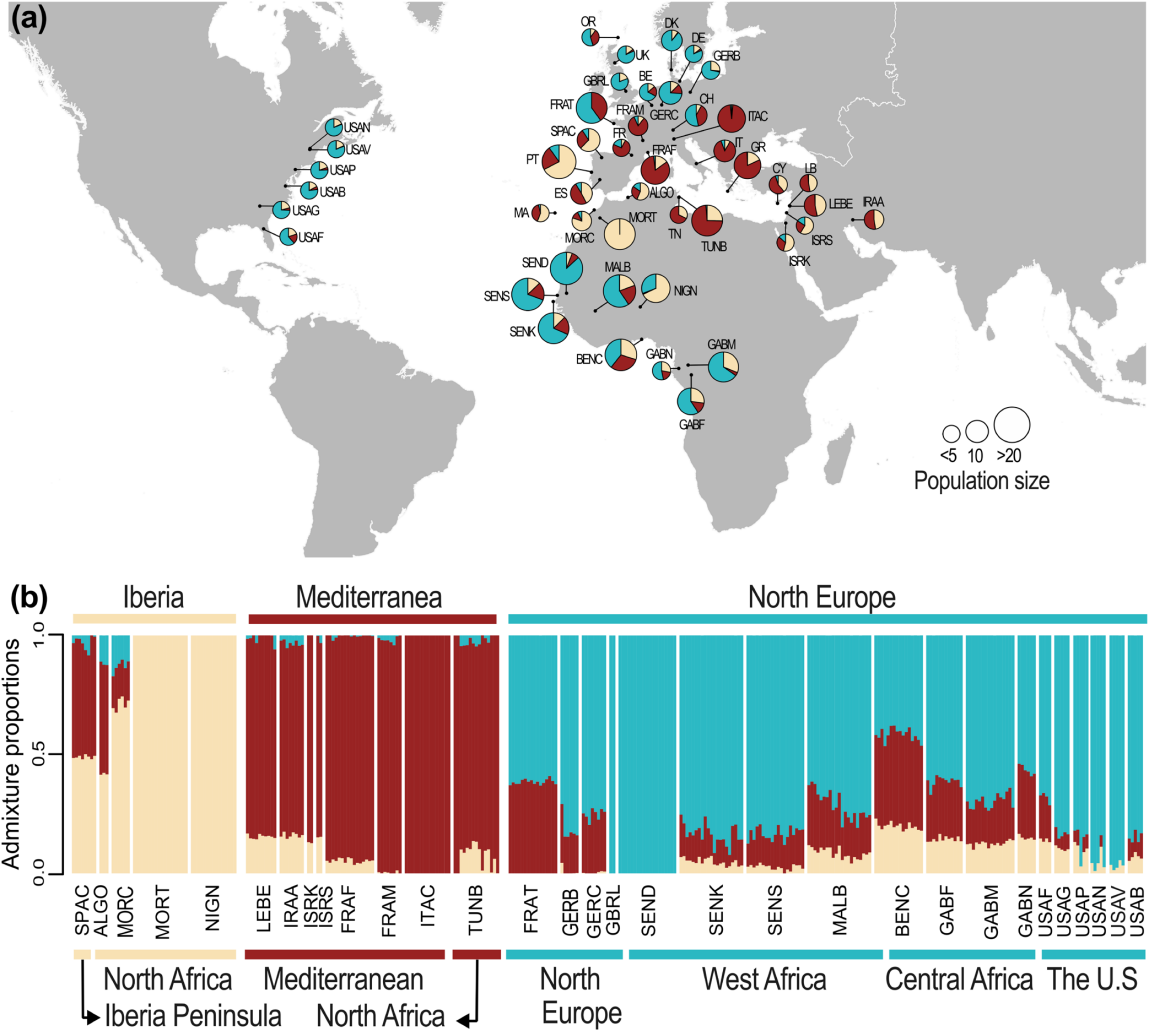
Spatial distribution of genetic ancestry of western house mouse populations. (a) Geographic distribution of the three major genetic lineages in the merged house mouse dataset based on 471 individuals. Pie charts represent the mean admixture proportions of individuals sampled at each location, estimated using sNMF. (b) Admixture proportions estimated using NGSadmix on 317 unrelated *M. m. domesticus* genomes, assuming three ancestral populations. Top labels indicate inferred ancestral clusters, while bottom labels correspond to sampled populations. Both analyses support at least three independent colonisation events of the western house mouse into Africa from Iberian (Morocco and Algeria in North Africa, and Niger), Mediterranean (Tunisia in North Africa), and Northern European sources (West and Central Africa populations). Population labels are shown for all sampling locations. Four‐letter population codes correspond to whole‐genome sequencing (WGS) populations included in both panels (a) and (b), whereas two‐letter population codes indicate populations genotyped using SNP arrays from Morgan et al. (2022) included only in panel (a). While the sNMF (a) and NGSadmix (b) analyses were performed on distinct datasets, they reveal congruent large‐scale ancestry patterns among African and Eurasian *M. m. domesticus* populations. Additional ancestry solutions for higher *K* values (*K* = 2–9) are shown in Figure S4 (NGSadmix) and Figure S5 (sNMF).

We further inferred population structure by estimating individual ancestry proportions under varying numbers of ancestral populations (K), using NGSadmix in the western house mouse dataset and sNMF in the merged dataset, which both yielded consistent results. At *K* = 3, ancestry components closely mirrored PCA patterns (Figure 2a,b), supporting at least three independent colonisation events of Africa: (i) populations from Morocco, Algeria and Niger shared predominant Iberian ancestry; (ii) Tunisian mice exhibited mainly Mediterranean ancestry with minor Iberian input; (iii) Senegal, Mali, Gabon and Benin populations shared mostly Northern European ancestry, with varying Mediterranean and Iberian contributions. Benin shows more mixed ancestry across all three components. North American populations align most closely with Senegalese mice, with additional Iberian and Mediterranean inputs, especially in Florida, where admixture is the highest.

### 3.2 Gene Flow Between the Western House Mouse and the Algerian Mouse

**FIGURE 3:**
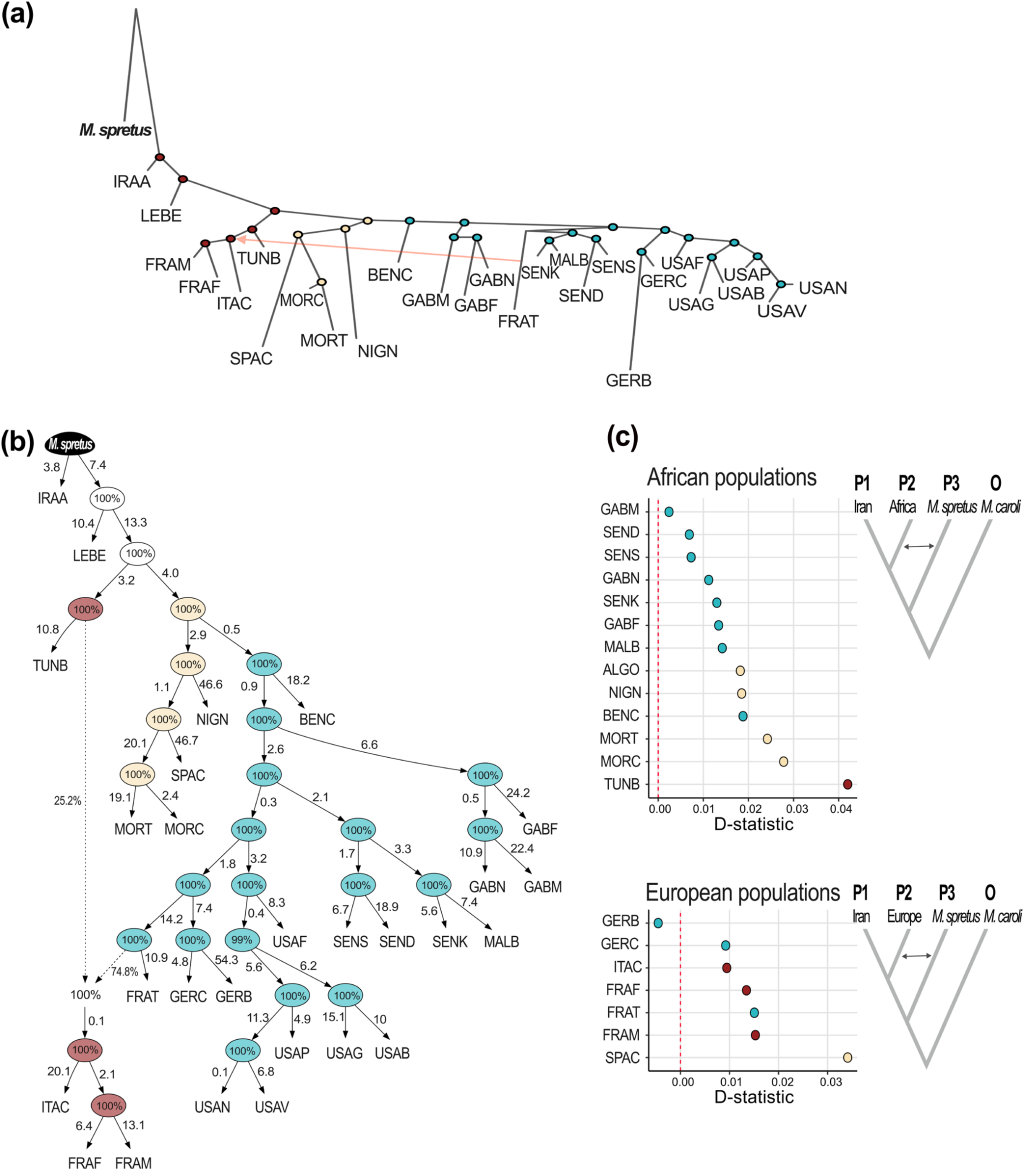
Population admixture and introgression. (a) Population tree inferred with TreeMix using the optimal number of migration edges (m = 1), as determined by OptM. The single inferred admixture event connects the Mediterranean and Northern European clusters (orange arrow; migration weight = 0.545). Alternative topology for *m* = 0 to *m* = 3, together with the full OptM diagnostics, are provided in Figure S8. (b) Population graph with the highest posterior probability reconstructed using AdmixtureBayes. Branch lengths represent genetic drift (scaled ×100). Posterior probabilities indicate support for each node. The dotted arrow marks the inferred admixture event, with the accompanying value indicating the estimated admixture proportion. Both TreeMix and AdmixtureBayes independently recover the same single migration event. (c) Results of *D*‐statistics (Durand et al. 2011) testing for excess allele sharing between African (top panel) and European (bottom panel) *M. m. domesticus* populations (P_2_) and *M. spretus* (P_3_), relative to the Iranian *M. m. domesticus* population (P_1_; IRAA). *M. caroli* was used as the outgroup. The statistic is constructed as *D* (P_1_, P_2_, P_3_, outgroup), where significantly positive values indicate gene flow between P_2_ and P_3_ relative to P_1_. Analyses were performed genome‐wide using all variable sites. All estimates were statistically significant, except for the comparison involving Berlin in Germany (GERB) (Table S3). Population codes correspond to those in Figure 1a.

To further reconstruct the colonisation history of African house mice while accounting for population admixture events between populations, we applied TreeMix and AdmixtureBayes. Both methods confirmed the major clusters identified by PCA and admixture analyses (Figure 3a,b). West Asian populations appeared most ancestral, while African populations clustered with European lineages, supporting at least three independent invasion events from Eurasia. Tunisia grouped with Italy and southern France (Mediterranean cluster), Morocco and Niger with Spain (Iberian cluster), while the other African populations (Senegal, Gabon, Mali, Benin), along with North American samples, clustered with Germany and northern France (Northern European cluster). Using TreeMix and selecting the optimal number of migration edges with OptM (*m* = 1; Figure S6), we detected a single admixture event between the Mediterranean and Northern European clusters (Figure 3a). AdmixtureBayes recovered an equivalent graph with one admixture edge linking the same European lineages (Figures 3b and S7). When allowing additional migration edges in TreeMix, the overall topology remained stable across models, including the mixture‐free tree (*m* = 0). Under these models, TreeMix inferred a signal of gene flow between *M. spretus* and the African population from Mali (MALB) at *m* = 2, and a second signal between *M. spretus* and the African population from Benin (BENC) at *m* = 3 (Figure S6). Given the present‐day geographic distribution of *M. spretus* , these edges likely reflect historical introgression rather than recent migration. Together, these patterns are consistent with the possibility of introgression between the house mouse and *M. spretus* in Africa.

To investigate further introgression between African house mice and native *M. spretus* , as previously reported in Europe (Banker et al. 2022), we then computed *D*‐statistics (Durand et al. 2011) with the Asia‐restricted species *Mus caroli* as outgroup. All African populations of the western house mouse showed excess allele sharing with *M. spretus* , with the signal decreasing from north to south (Figures 3c and S8). A similar pattern was evident in European mice, with allele sharing decreasing from the southernmost (Spain and France) to the northernmost (Germany) populations; only one German population (Berlin) showed no detectable introgression. Notably, admixture in Spain, within the sympatric range of both species, was twice as high as in other European populations. Conversely, North American populations did not show any signal of introgression with *M. spretus* (Table S3). Estimates of the admixture proportion (*f*) further quantified variation in *M. spretus* allele sharing among African and European populations. The highest estimated admixture proportion was detected in Tunisia (TUNB, *f* = 4.5%, 95% CI: 4.08%–4.92%), followed by coastal Morocco (MORC, *f* = 2.98%, 95% CI: 2.56%–3.40%), whereas Spain showed the strongest signal within Europe (SPAC, *f* = 3.67%, 95% CI: 3.11%–4.23%) (Table S3). These results support significant but geographically heterogeneous introgression from *M. spretus* into *M. m. domesticus*, particularly in North Africa and in European regions of sympatry, contributing to the complex genomic mosaic of African house mice.

### 3.3 Demographic History and Colonisation of the House Mouse in Africa

**FIGURE 4:**
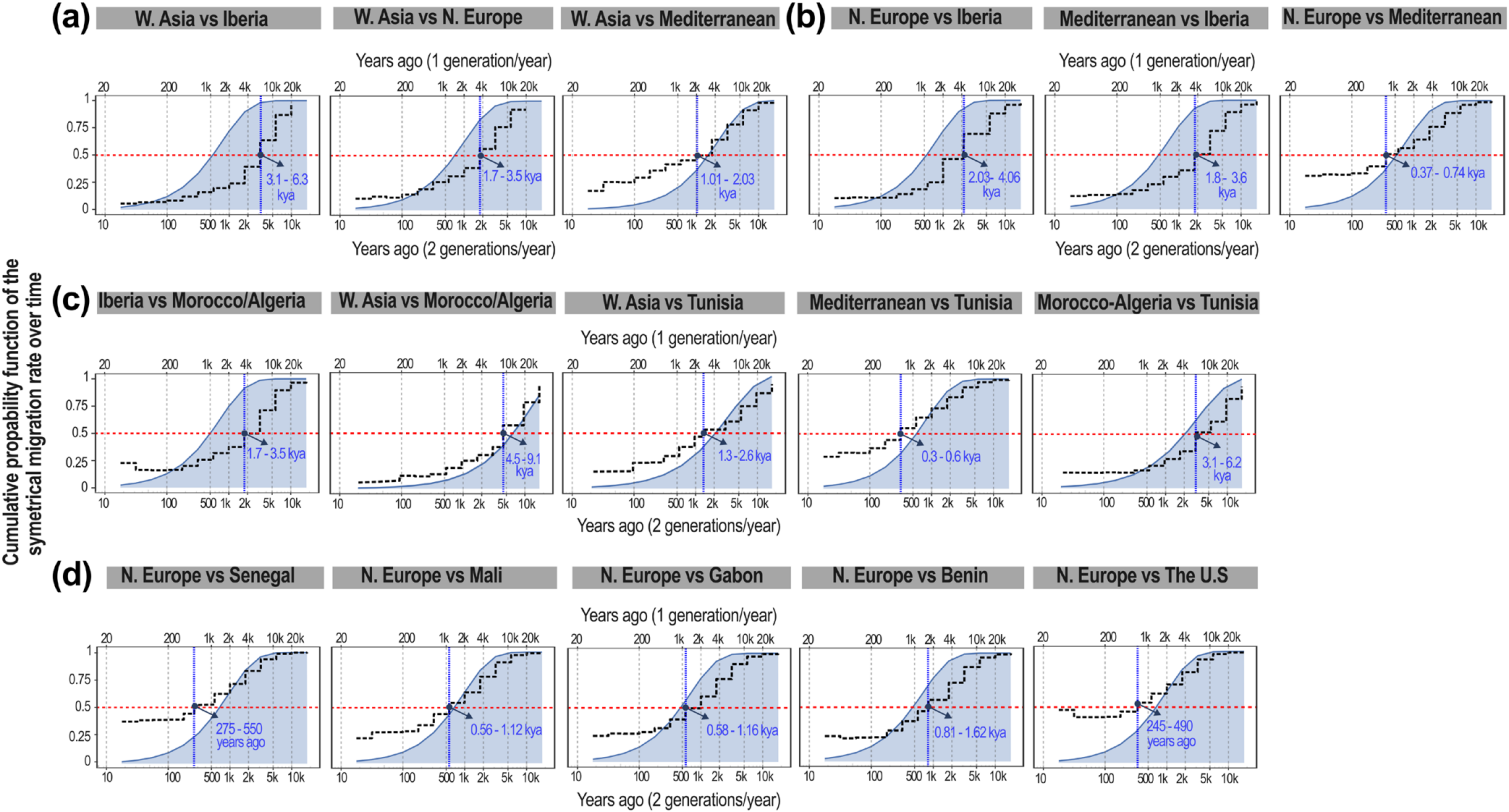
Divergence times inferred from cross‐coalescence rates in the house mouse. Divergence between the indicated population pairs was estimated using cross‐coalescence rates inferred from Relate and modelled under the Multiple Sequentially Markovian Coalescent—Isolation with Migration model (MSMC‐IM). (a) Divergence between West Asian populations and the three major European *M. m. domesticus* lineages. (b) Divergence among the three major European lineages. (c) Divergence between North African populations and their putative Eurasian sources (d) Divergence between Northern European populations and derived populations from West Africa, Central Africa, and North America. Dashed black lines show the relative cross‐coalescence rate (rCCR) through time (*x*‐axes are log_10_‐transformed). The red dashed vertical line marks the inferred split time, defined as the point at which rCCR declines to 0.5, corresponding to the midpoint of lineage separation. Blue shaded areas represent the cumulative distribution of migration probabilities over time, reflecting the proportion of shared ancestry already merged at a given time point. Values approaching 0 indicate near‐complete isolation, whereas values approaching 1 indicate high genetic continuity between groups. All estimates assume a mutation rate of 4.1 × 10^−9^ per site per generation (Geraldes et al. 2011). The upper *x*‐axis shows time assuming one generation per year (Geraldes et al. 2008), whereas the lower *x*‐axis assumes two generations per year (Bronson 1979), illustrating the sensitivity of inferred divergence times to generation‐time assumptions.

One objective of this study was to determine the timing of house mouse population separation during its expansion into Africa. We first examined separation among the major *M. m. domesticus* lineages and their separation from West Asian populations (Figure 4a,b). CCR trajectories indicate a hierarchical pattern of declining connectivity with West Asia. The Iberian lineage shows the earliest reduction in shared ancestry with West Asia (CCR < 0.5 at ~3.1–6.6 kya, assuming two different generation time assumptions), followed by Northern Europe (~1.7–3.5 kya), and subsequently the Mediterranean lineage (~1.01–2.03 kya). The split between Northern Europe and Iberia is estimated at approximately ~2.03–4.06 kya, between the Mediterranean and Iberia at ~1.8–3.6 kya, and between Northern Europe and the Mediterranean more recently, at ~0.37–0.74 kya (Figure 4b).

The inferred separation process between European and African populations was generally recent, with the notable exception of African populations carrying Iberian ancestry (Figure 4c). For North African populations from Morocco and Algeria, separation from Iberia is estimated at approximately 1.7–3.5 kya. In contrast, Tunisia, which carries predominantly Mediterranean ancestry, shows a more recent split from Mediterranean Europe, estimated at approximately 0.30–0.60 kya. We also examined separation between West Asia and North African populations, and within North African populations. Morocco–Algeria shows early separation from West Asia (approximately 4.5–9.1 kya), whereas Tunisia separated from West Asia approximately 1.3–2.6 kya. The split between Morocco‐Algeria and Tunisia occurred approximately 3.1–6.2 kya. Finally, for West and Central African populations, as well as North America, separation from Northern Europe is inferred to have occurred recently (Figure 4d). Estimated split times are approximately 0.28–0.55 kya for Senegal, 0.56–1.12 kya for Mali, 0.59–1.16 kya for Gabon, and 0.81–1.62 kya for Benin. North American populations show even more recent separation from their Northern European source, estimated at approximately 0.24–0.49 kya. We further explored divergence among West and Central African populations and divergence between Niger and North African populations (Morocco‐Algeria). However, in several of these comparisons, the CCR did not reach the 0.5 threshold (e.g., Senegal versus Mali), or the inferred split times were substantially deeper, suggesting a limited resolution for these divergence events (Figure S9). These patterns indicate limited resolution of CCR‐based separation estimates for very recent divergence events and suggest that some inferred split times likely reflect older demographic signals inherited from source populations rather than recent in situ divergence within Africa.

**FIGURE 5:**
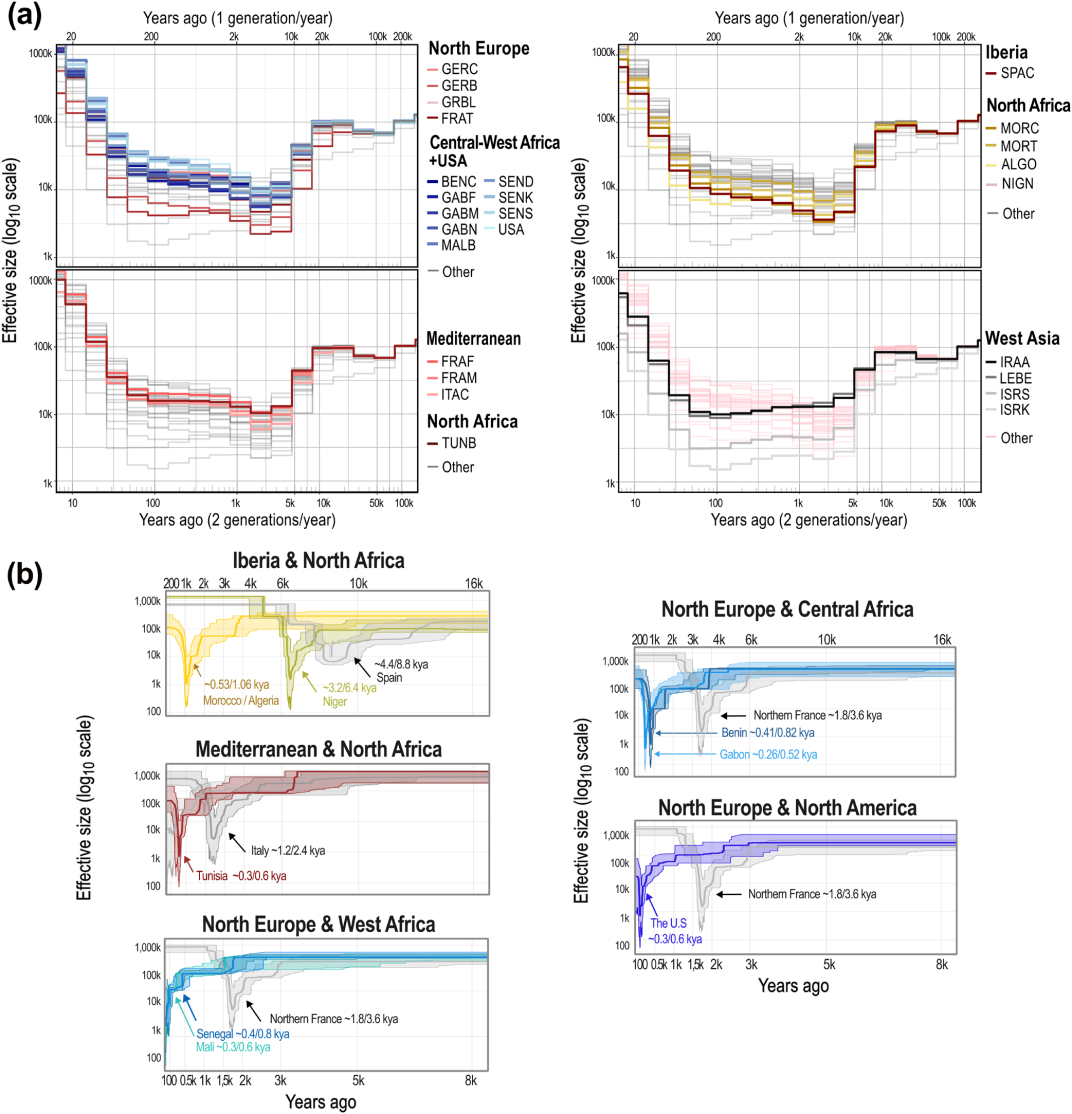
Changes in effective population sizes. (a) Inferred effective population size (*Ne*) trajectories over time for 357 imputed *M. m. domesticus* genomes using Relate and genome‐wide genealogies. Each subpanel highlights populations belonging to a given ancestry group, while remaining populations, denoted as ‘Others,’ are shown in the background for comparison. (b) Inferred effective population size of representative populations (*n* > 10) inferred from a genome‐wide SFS using Stairway Plot 2. Populations from the same region sharing similar ancestry profiles were intentionally merged to improve SFS estimation. This approach was applied to Senegalese, Gabonese, and North American populations, which were grouped based on their respective ancestry profiles inferred from population structure analyses (Figure 2). Solid lines represent median *Ne* estimates, and shaded areas indicate 95% confidence intervals. African populations (coloured) are compared to the European ones from the same ancestry, indicated above each panel. Grey lines depict the *Ne* estimates for the corresponding European populations. In both panels (a) and (b), the upper *x*‐axis shows time assuming one generation per year (Geraldes et al. 2008), whereas the lower *x*‐axis shows time assuming two generations per year (Bronson 1979). This dual representation illustrates the sensitivity of *N*_*e*_ trajectories to assumptions about generation time and provides a range of plausible temporal interpretations. All estimates assume a mutation rate of 4.1 × 10^−9^ per site per generation (Geraldes et al. 2011).

Relate‐based *N*_*e*_ reconstructions indicate that European, African, and North American populations exhibited broadly similar long‐term demographic histories, with overlapping *N*_*e*_ trajectories extending back approximately 100–200 kya (Figure 5a). A population decline was inferred beginning roughly 10–20 kya, with the most pronounced reduction occurring around 2.5–5 kya. Across populations, *N*_*e*_ subsequently increased during the last ~1.5–3 kya, consistent with post‐bottleneck demographic expansion. Notably, African and North American populations did not show clear evidence of additional recent contractions associated with their secondary colonisation from Europe or West Asia. Sensitivity analyses using alternative mutation rates (*μ* = 4.1 × 10^−9^ and *μ* = 5.7 × 10^−9^) showed that varying *μ* uniformly rescales the time and *N*_*e*_ axes but does not alter the overall demographic trajectories or the relative ordering of events (Figure S10).

**FIGURE 6:**
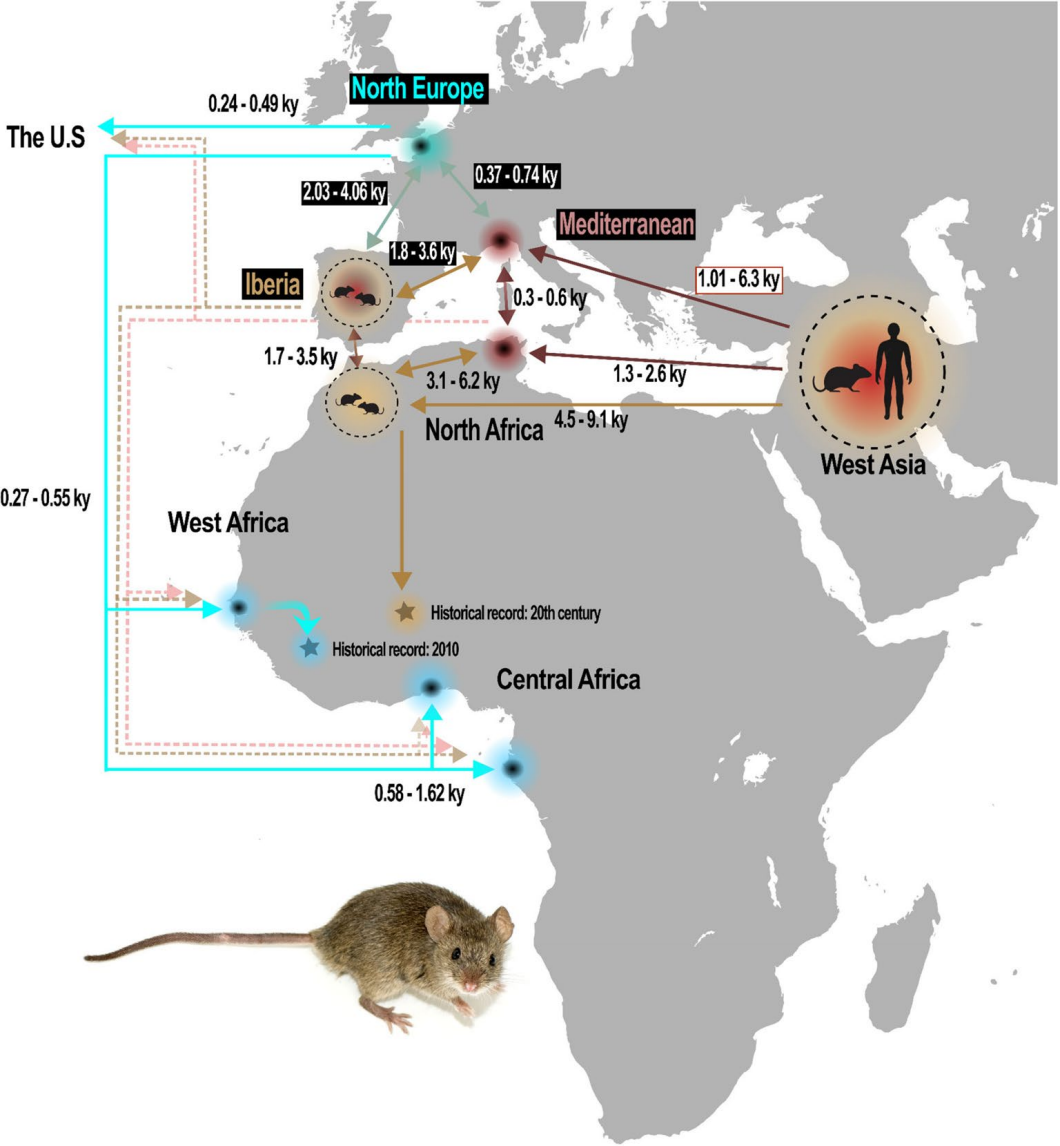
Proposed invasion dynamics of *M. m. domesticus* in Africa. Summary of cross‐coalescence rates (CCR) based separation patterns and inferred expansion routes of the house mouse from West Asia to Europe, Africa, and North America. Time ranges adjacent to arrows represent CCR‐based separation estimates (in thousand of years, ky) obtained using MSMC‐IM, defined as the time at which the CCR declines to 0.5. Separation ranges of the three major European lineages from West Asia is inferred to have occurred between ~1.0 and ~6.3 kya, consistent with westward expansion likely facilitated by human dispersal (indicated by human and mouse icons). Within Europe, lineage separations are estimated between ~0.37 and ~4.06 kya. The expansion into Africa involved multiple European and/or West Asian sources. Relatively deep coalescent separation signals are observed between West Asia and Morocco–Algeria (~4.5–9.1 kya) and between Morocco–Algeria and Tunisia (~3.1–6.2 kya), supporting early lineage branching in western North Africa during westward expansion. By contrast, the recent Tunisia–Mediterranean Europe separation (~0.3–0.6 kya) likely reflects shifts in effective connectivity within a historically interconnected western Mediterranean system. West and Central African populations show separation from Northern Europe between ~0.27 and 1.6 kya, consistent with introduction during the period of European maritime expansion. Finally, North American populations show separation from Northern Europe at ~0.24–0.49 kya, consistent with historical colonisation. Dashed arrows illustrate additional ancestry contributions from Mediterranean and Iberian lineages into West and Central Africa and North America, as suggested by admixture profiles showing mixed European ancestry components. Where CCR‐based estimates lacked resolution for recent events, historical records are indicated with a star (e.g., Niamey, late 20th century; Bamako, likely 2010s introduction from Senegal). Significant introgression between native *M. spretus* and *M. m. domesticus* is observed in Europe (particularly the Iberian Peninsula) and in North Africa, represented by two mouse schematic icons. All time ranges are scaled assuming *μ* = 4.1 × 10^−9^ per site per generation and alternative generation‐time assumptions (see Methods), House mouse photo from Wikimedia Commons by George Shuklin, licensed under CC BY‐SA 1.0.

Stairway Plot analysis revealed successive *N*_*e*_ declines across European populations, consistent with the expansion of *M. m. domesticus* out of West Asia (Figures 5b and S11). In Spain, the main decline reached its minimum at ~4.4–8.8 kya; in Italy at ~1.2–2.4 kya; and in southern France at ~0.3–0.6 kya under alternative generation‐time scalings. In Northern Europe, the demographic minimum occurred at ~1.8–3.6 kya in northern France and ~0.6–1.2 kya in Germany. In North Africa, the demographic trajectories of Morocco and Algeria reveal two marked *N*_*e*_ reductions (Figure 5b). The first decline occurred at ~1.7–3.4 kya, followed by a pronounced recent bottleneck peaking approximately at ~0.5–1.07 kya, during which *N*_*e*_ dropped to ~1000 before recovering to ~100,000. In Niger, we detect an older bottleneck inferred at ~3.2–6.4 kya, with no evidence of more recent contraction. Tunisia shows a strong bottleneck at ~0.3–0.6 kya, after which *N*_*e*_ recovered to ~70,000. In Central Africa (Gabon and Benin), we identify a strong recent bottleneck peaking at ~0.26–0.52 kya in Gabon and ~0.41–0.82 kya in Benin. In both populations, *N*_*e*_ subsequently increased to roughly ~110,000. In West Africa, populations from Senegal and Mali exhibit highly similar trajectories, with a pronounced decline within the last ~0.3–0.8 kya. The North American population experienced a parallel demographic history, characterised by a strong *N*_*e*_ reduction during the past ~0.3–0.6 kya, followed by a recovery to approximately 20,000 *N*_*e*_. Overall, these patterns support a two‐step demographic history (Figure 6): (i) an ancient bottleneck associated with the out‐of‐West‐Asia expansion of *M. m. domesticus*, and (ii) region‐specific recent bottlenecks, consistent with multiple, independent introductions into North, West, and Central Africa, as well as North America.

### 3.4 Genomic Diversity and Inbreeding in African House Mice

**FIGURE 7:**
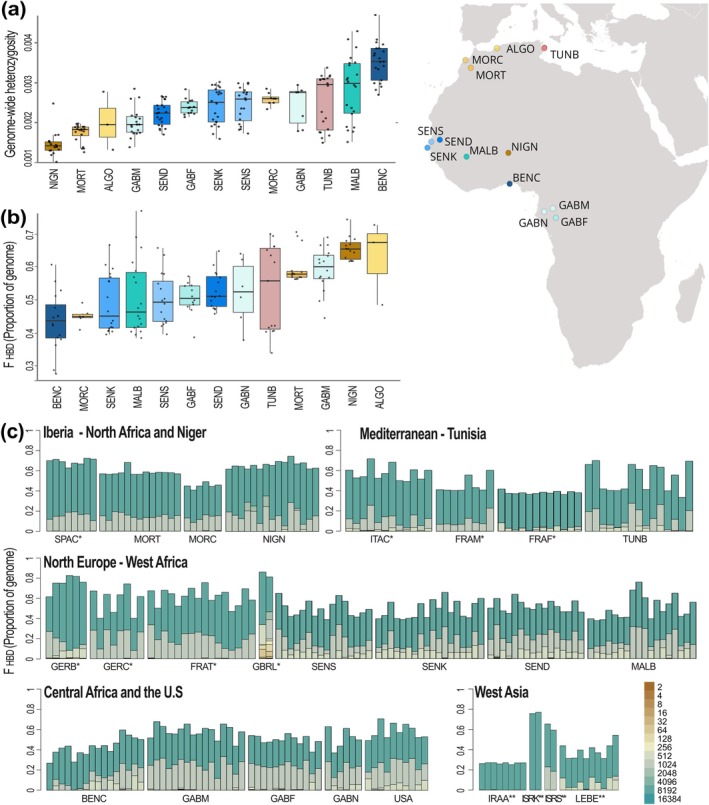
Genome‐wide heterozygosity and inbreeding patterns in African house mouse populations. (a) Genome‐wide heterozygosity, measured as the proportion of heterozygous sites per base pair, shown for individual mice and stratified by sampling location across Africa. (b) Realised inbreeding coefficient (*F*_HBD_), where HBD denotes homozygous‐by‐descent, estimated for each population using RZooRoH from posterior HBD probabilities. *F*_HBD_ reflects the proportion of the autosomal genome in homozygous‐by‐descent states and captures variation in recent versus ancient inbreeding. Populations are grouped geographically and ordered by ascending mean *F*_HBD_. Boxplots show the median (center line), interquartile range (box), and 1.5× IQR whiskers. (c) Partitioning of individual genomes into homozygous‐by‐descent (HBD) classes of different ages using RZooRoH. Each stacked bar represents one individual, with colours corresponding to HBD classes. Older HBD classes (high 𝑅_𝑘_) denote ancient ancestry, whereas younger classes indicate more recent inbreeding. Populations show contrasting profiles, with West Asian individuals dominated by ancient HBD classes, European mice showing a mix of ancient and intermediate classes, and West and Central African and North American populations exhibiting an additional enrichment of younger HBD classes following colonisation. (*) denotes European populations.

Patterns inferred from heterozygosity, nucleotide diversity, and Watterson's *θ* were highly concordant across populations (Figure S12); hereafter, we focus on heterozygosity as a summary of overall diversity and use Tajima's *D* to describe complementary differences in the frequency of rare and intermediate‐frequency variants. Across Africa, heterozygosity varied substantially among populations, ranging from low values in Niger (0.0010) and in some inland Moroccan populations (e.g., MORT, 0.0012) to relatively high values in Mali (0.0030) and Benin (0.0036) (Figure 7a). When extending the comparison beyond Africa, West Asian populations exhibited some of the highest levels of heterozygosity overall, particularly in Iran (0.0045) and Lebanon (0.0038). Comparisons among populations sharing the same ancestry revealed contrasting patterns. Within the Iberian cluster, populations from Morocco and Algeria displayed higher heterozygosity than the population from Spain. Within the Mediterranean cluster, southern France (FRAF) showed slightly higher heterozygosity than Tunisia, whereas inland populations in France and Italy exhibited a marked reduction in heterozygosity. Within the Northern European cluster, Northern European populations generally showed similar or even lower heterozygosity compared to derived populations from West and Central Africa and North America (Figure S12). This pattern was particularly pronounced in Benin and Mali in Africa and in Florida in North America (USAF), which displayed among the highest heterozygosity values and also showed admixed ancestry profiles (Figure 2). Tajima's D provided a complementary view of the demographic processes underlying these diversity patterns (Figure S12). Populations from the native West Asian range showed values close to neutrality or slightly negative, as observed in Iran, whereas several populations in the invasive range showed positive Tajima's *D* values. Moroccan and Algerian populations showed generally positive Tajima's *D*, consistent with an excess of intermediate‐frequency variants that may reflect demographic contraction, drift, population structure, or admixture rather than recent expansion alone. Niger also combined low heterozygosity with strongly positive Tajima's *D*, supporting a scenario of reduced diversity and strong drift and/or bottleneck effects. In contrast, Mali showed relatively high heterozygosity but negative Tajima's *D*, suggesting an excess of rare variants compatible with recent expansion or connectivity after introduction. Among highly diverse admixed populations, Benin showed high heterozygosity with Tajima's *D* values close to neutral or slightly positive, whereas the North American Florida population showed negative Tajima's *D* in our dataset. Overall, Tajima's *D* indicates that elevated diversity in invasive populations can arise through different combinations of source admixture, demographic contraction, and post‐introduction expansion, reinforcing that African house mouse populations followed distinct demographic trajectories after colonisation. We identified 3,909,512 HBD segments across African populations, indicating contributions from both recent and ancient inbreeding. The realised inbreeding coefficient (*F*_HBD_) varied substantially among populations (ANOVA: *F*_12,159_ = 7.63, *p* = 4.6 × 10^−11^) and differed significantly between inland and coastal regions (ANOVA: *F*_1,152_ = 6.79, *p* = 0.01). Across African populations, *F*_HBD_ values ranged from 0.27 to 0.77 (Figure 7b). Coastal populations, including Benin (BENC, 0.43), Morocco (MORC, 0.45), and Senegal (SENK, 0.49), showed the lowest inbreeding levels, consistent with higher genetic diversity and suggesting weaker recent founder effects, possibly due to greater connectivity. In contrast, inland populations exhibited markedly higher *F*_HBD_, particularly Niger (NIGN, 0.66), Gabon (GABM, 0.59), and inland Morocco (MORT, 0.60), indicative of strong drift or persistent demographic isolation. Mali (MALB), however, represents a notable exception: despite being inland, its *F*_HBD_ levels resemble those of coastal populations, suggesting substantial recent connectivity compared to neighbouring inland regions.

Across all regions, genomes were dominated by the oldest HBD classes (𝑅 = 16,384), corresponding to approximately 8192 generations (with HBD age approximated as ½ × 𝑅). This translates to roughly ~4096 years assuming two generations per year, or ~8192 years assuming one generation per year. These older HBD classes reflect deep shared ancestry predating the recent global expansion of *M. m. domesticus* (Figure 7c). This ancestry signal was strongest in West Asian populations, which showed almost exclusively ancient HBD contributions. European mice retained this ancestral background but also exhibited intermediate‐age classes (𝑅 = 1024; ~256 and ~512 years), and two isolated European populations (Germany and England) displayed very recent HBD classes consistent with strong contemporary inbreeding. African populations retained the ancient HBD component present in Europe and West Asia (𝑅 > 1024), but West and Central African populations, as well as the North American population, showed a pronounced enrichment of younger HBD classes, particularly 𝑅 = 512 (~128 and ~256 years), that are rare or absent in Northern European mice. This pattern is consistent with post‐colonisation inbreeding driven by strong founder effects and local isolation. Spain, Morocco, and Niger were characterised by predominantly ancient HBD classes, with limited contributions from younger classes. Mediterranean populations showed highly homogeneous profiles.

## 4 Discussion

Here, we present 303 newly generated whole‐genome individual sequencing data representing *M. m. domesticus* populations sampled across West Asia, Europe and Africa, combined with 77 previously published sequenced genomes in order to depict the colonisation history, population structure, interspecific gene flow as well as genetic diversity of the invasive western house mouse in Africa. Our analyses provide an unprecedented resolution on the demographic events accompanying house mouse introduction in Africa, and refine existing models of their expansion into Europe and North America. In Africa, we reveal that the invasion was not a single demographic process, but a mosaic of population‐specific histories shaped by multiple independent colonisation events from distinct Eurasian sources, strong population bottlenecks, secondary admixture, and introgression with *M. spretus* in North African populations. Heterogeneous patterns of heterozygosity, Tajima's D, and genomic inbreeding further show that African populations experienced different combinations of founder effects, connectivity, drift, and admixture. These results provide new insight into how human‐mediated dispersal and secondary contact with native species can shape the genomic legacy of an invasive commensal mammal, and provide a valuable resource for future studies on invasion genomics, hybridisation, and rapid adaptation.

We identify three major *M. m. domesticus* lineages in Europe, all of which are represented in African mouse populations. The inferred separation of these lineages from West Asian populations over approximately ~1–6 kya, followed by diversification within Europe occurring broadly between ~0.4 and ~4 kya is consistent with a westward expansion from West Asia, subsequent regional differentiation across Europe, and later episodes of connectivity (Figure 6). These estimates broadly align with previous genomic studies (Morgan et al. 2022; Agwamba et al. 2025), and are compatible with archaeological evidence indicating that house mice colonised Europe during the Bronze Age (Cucchi et al. 2020). However, some inferred dates in our analyses are shifted toward more recent values compared with previous estimates, including the West Asia–Europe separation and the Northern European–Mediterranean split. We interpret these differences cautiously because previous studies generally estimated divergence under stricter bifurcating models (Agwamba et al. 2025), whereas the MSMC‐IM model used in our study does not infer a strict split time. Instead, it estimates changes in effective cross‐coalescence through time and can accommodate post‐divergence gene flow. In the presence of ongoing or recent connectivity, CCR‐based estimates may therefore be shifted toward more recent dates, reflecting changes in gene flow rather than the initial onset of lineage separation (Wang et al. 2020). This interpretation is particularly relevant for geographically close and historically connected lineages, such as West Asian and Mediterranean populations, and may also explain the recent Northern European–Mediterranean separation inferred here, which is consistent with post‐divergence connectivity supported by TreeMix and AdmixtureBayes.

In North Africa, our results support at least two independent introduction histories; one in Tunisia carrying predominantly Mediterranean ancestry, and another in Morocco and Algeria carrying Iberian‐related ancestry. These patterns suggest that the western Mediterranean radiation of *M. m. domesticus* involved multiple closely spaced lineage splits and episodes of connectivity, rather than a simple stepwise dispersal from Europe into Africa. The deeper coalescent signals observed in Morocco and Algeria, together with their relatively high heterozygosity compared with some European populations, argue against a simple recent Europe‐to‐Africa founder scenario. Instead, they are compatible with western North Africa participating early in the broader western Mediterranean radiation, potentially concurrently with, or close in time to, southern Europe. This genomic interpretation aligns with zooarchaeological debates regarding dispersal routes. While the prevailing hypothesis emphasises an east‐to‐west expansion along the Mediterranean coast (Cucchi et al. 2005; Bonhomme et al. 2011), Cucchi et al. (2005) also reported remains attributed to *Mus musculus* from the Canary Islands dated to ~7000 years ago. They proposed that, if these prehistoric specimens indeed derived from mainland house mouse populations, dispersal may have followed an ‘African route’ operating in parallel to the Mediterranean coastal pathway, potentially bringing house mice into parts of North Africa as early as the 7th millennium BC. Although the fossil record remains sparse and interpretations debated, our deeper CCR‐based signals in North Africa are compatible with such an early African‐associated pathway. We caution that genetic divergence times do not necessarily correspond to the first archaeological appearance of house mice in a given region. For example, the recent Tunisia–Mediterranean split (~0.3–0.6 kya) likely reflects shifts in effective connectivity within a region characterised by sustained maritime exchange and commercial interaction (Oliver and Atmore 2001; Abulafia 2011; Abun‐Nasr 1987).

Demographic reconstructions further suggest that North African populations experienced population contractions after their initial establishment. In Morocco and Tunisia, bottleneck estimates broadly overlap with the late medieval to early modern period, a time of political fragmentation, urban restructuring, and changing trade connectivity across North Africa (Oliver and Atmore 2001). Although genomic data cannot establish direct causation, such transformations may have influenced commensal rodent populations through changes in settlement density and trade connectivity. Together, the demographic evidence supports a dynamic western Mediterranean history characterised by early lineage diversification, parallel dispersal routes, and subsequent bidirectional exchanges across the Mediterranean basin. Further south, mice from Niger show strong genetic affinity with Moroccan populations. While this pattern could reflect older trans‐Saharan connections (Lovejoy 2000; Henn et al. 2012; Harich et al. 2010), several lines of evidence point instead to a more recent and restricted establishment. Niamey is a relatively recent urban center, and house mice were only recorded there toward the end of the 20th century (Garba et al. 2014). Consistent with this scenario, the Niger population combines low genetic diversity, strong genomic inbreeding, and positive Tajima's *D*, a pattern compatible with strong drift and/or bottleneck effects after the introduction of a limited number of founders. This suggests that contemporary or recent trans‐Saharan commercial movements may have contributed to the establishment of this population.

In West and Central Africa, *M. m. domesticus* populations predominantly exhibit Northern European ancestry (Figure 2), consistent with introductions linked to European maritime expansion and colonial activity. Divergence and bottleneck estimates broadly overlap with the period of early European coastal trade and subsequent colonial expansion along the West African coast (Nicolle 2012; Newitt 2004; Sinou 1993). In Senegal, the timing of divergence from Northern Europe and local bottlenecks is compatible with the establishment of permanent trading posts and colonial cities, including Saint‐Louis, founded by the French in 1659 and later occupied by the British in 1693 (Sinou 1993). The presence of mice in Bamako (Mali) is likely very recent (2010s), probably originating from Senegal (Ag Atteynine et al. 2026). The divergence inferred in Mali may therefore reflect a demographic history already present in the source population, rather than one occurring after establishment in Mali. In contrast, the port of Cotonou in present‐day Benin appears to have facilitated multiple introductions from European sources, likely associated with historical coastal trade and subsequent inland movements, resulting in highly admixed mouse population.

As in West African mice, North American house mice predominantly descend from Northern European lineage. Florida populations, in particular, display an admixed genetic profile. Demographic bottleneck and divergence time estimates align with later phases of European colonisation, when commercial exchanges between regions intensified. Both results support the hypothesis of dual introductions via Spanish and British routes (Agwamba and Nachman 2023). Spanish expeditions to Florida, followed by British control, likely facilitated successive waves of mouse introductions. This pattern contrasts with some island systems, where historical British maritime influence likely contributed to the predominance of Northern European ancestry, but geographic isolation and small founder sizes may have constrained subsequent admixture despite maritime connections (Hardouin et al. 2010; Morgan et al. 2022). Taken together, these findings underscore how layered waves of European maritime contact, colonial expansion, and inland trade shaped regionally distinct colonisation histories of house mice across West and Central Africa and North America, whereas geographic isolation may have constrained the impact of repeated introductions in some island systems. The recurring signal of mixed ancestry, from coastal Africa to the eastern United States, highlights the complexity of these dispersal routes and the lasting genetic legacy of European colonial movements on house mouse diversity across continents.

A note of caution is warranted before drawing definitive historical conclusions. Demographic inference is inherently sensitive to assumptions about mutation rate, generation time, population structure, and gene flow. Varying the mutation rate or generation time mainly rescales the time axis and *Nₑ* values, whereas admixture among European lineages or during African colonisation can blur the apparent timing of population separations and bottleneck events (Speidel et al. 2019; Liu and Fu 2020). Furthermore, because our analyses rely on low‐coverage whole‐genome data, genotype imputation may introduce additional uncertainty despite stringent filtering and validation against high‐coverage genomes. Consequently, inferred demographic dates should be interpreted as coarse estimates rather than precise historical timestamps. Our sensitivity analyses under alternative mutation rates (*μ* = 4.1 × 10^−9^, 5.7 × 10^−9^, and 6.0 × 10^−9^; Figure S10) confirm that changing *μ* mainly rescales the time axis and *Nₑ* but does not alter the overall shape or relative ordering of demographic events. Using *μ* = 4.1 × 10^−9^, German and French populations exhibit a decline beginning ~15 kya, with bottleneck minima around ~2.5–5 kya. These recent bottlenecks should not be interpreted as contradicting older reductions in effective population size reported around the Last Glacial Maximum in previous studies, but rather as more recent lineage‐ or population‐specific contractions superimposed on a longer‐term decline. This interpretation is broadly consistent with previous genomic analyses (Ullrich and Tautz 2020; Chen et al. 2025; Agwamba et al. 2025), which also inferred Holocene population size reductions during the last few millennia. In addition, although our sampling represents the broadest genomic coverage of wild *M. m. domesticus* in Africa to date, continental coverage remains incomplete. Current data suggest that house mice from East Africa have genetic affinities with *M. m. castaneus*, despite harbouring a mixture of *M. m. castaneus* and *M. m. domesticus* matrilines (Hardouin et al. 2015; Morgan et al. 2022). Thus, the absence of East African *M. m. domesticus* populations in our dataset partly reflects the currently limited evidence for established *M. m. domesticus* populations in these regions. Nevertheless, more extensive sampling from East and South Africa will be required to test this explicitly and to fully resolve continental‐scale colonisation routes. Overall, the general patterns of population contraction, expansion, and divergence remain robust across parameter choices and align closely with known phases of maritime contact and colonial expansion, supporting the broader historical framework we propose.

Across Africa, present‐day genomic diversity reflects not only founder events but also admixture and geographic isolation. Coastal populations like those in Tunisia and Morocco, among the earliest colonised, show intermediate heterozygosity, moderate inbreeding, and generally positive Tajima's D values consistent with long‐term establishment and the accumulation of drift and/or population structure rather than a simple recent expansion. In contrast, more isolated inland populations exhibit higher inbreeding, consistent with recent secondary expansions from coastal hubs, likely facilitated by stepwise movements along transport routes such as roads and railways (Dalecky et al. 2015). The Niger population, in particular, shows extreme inbreeding (*F*_HBD_ > *0.6*), and positive Tajima's *D*, a pattern consistent with strong isolation and drift, potentially following a severe contraction. These patterns also align with the social structure of *M. m. domesticus*, where limited female dispersal often promotes localised inbreeding (Macholán et al. 2012; Phifer‐Rixey and Nachman 2015). Interestingly, Northern European source populations do not uniformly exhibit higher genetic diversity than derived populations. In several cases, invasive populations display equal or even greater heterozygosity despite their more recent establishment, deviating from expectations under a simple serial founder model. This pattern suggests that multiple introductions and admixture among differentiated European lineages have reshaped genomic diversity in some African mainland regions. However, Tajima's *D* indicates that elevated heterozygosity can reflect different demographic processes across populations. Benin illustrates a case where high heterozygosity, multi‐source admixture, and Tajima's *D* values close to neutral or slightly positive are consistent with repeated introductions from differentiated sources, possibly linked to its central role in Atlantic trade networks (Law 1991). Florida also shows high heterozygosity and evidence of multiple European introductions (Agwamba and Nachman 2023), but its ancestry profile is more strongly dominated by a Northern European component, and its negative Tajima's *D* suggests a stronger excess of rare variants. Although Tajima's *D* cannot distinguish admixture from demographic growth on its own, this contrast suggests that elevated heterozygosity in Benin and Florida likely arose through different combinations of source admixture and post‐introduction demographic change. These cases show that admixture can offset diversity loss associated with founder events, highlighting that commensal invasion histories reflect not only bottlenecks and drift, but also diversity‐enhancing gene flow driven by human movement and trade.

Signals of introgression from the native species *M. spretus* are widespread among African *M. m. domesticus* populations, with the strongest evidence observed in Tunisia and Morocco (Figure 3c), regions where both species are currently sympatric or geographically close. Introgression signals decrease toward West and Central Africa, with the weakest signal in Senegal and Gabon. This geographic gradient mirrors patterns also observed in Europe, where *M. spretus* introgression is strongest in southern regions and weakens northward (Banker et al. 2022). However, disentangling the origins of these signals in Africa is challenging due to the continent's complex colonisation history. Our demographic analyses indicate at least three independent introductions of *M. m. domesticus* into Africa from genetically distinct European populations, each already carrying varying levels of *M. spretus* ancestry. Thus, the introgression signatures observed in Africa likely reflect either ancient gene flow inherited from already‐admixed European source populations or more recent hybridisation occurring locally in zones of current sympatry. While formally disentangling these components would require ancestry‐resolved analyses (e.g., haplotype‐based methods), our current genome‐wide approach provides strong evidence for a geographic gradient of introgression consistent with isolation‐by‐distance, where signals are strongest in North Africa and diminish toward the south. Notably, we detect no evidence of *M. spretus* introgression in North American populations, consistent with their predominant origin from Northern European lineages, which show limited introgression both in our data and in prior studies (Banker et al. 2022), and with the fact that *M. spretus* is not sympatric with *M. m. domesticus* in North America. This absence is consistent with the interpretation that *M. spretus* ancestry in African populations reflects a combination of inherited ancestry from introgressed source populations and, in North Africa, possible local gene flow within or near the native range of *M. spretus* . Future work incorporating ancestry‐resolved methods will be key to disentangling the timing of these introgressed segments. Introgression with *M. spretus* may have had functional and evolutionary consequences. In European *M. m. domesticus*, introgressed genomic regions are enriched for olfactory receptor genes and loci associated with environmental adaptation (Banker et al. 2022). Testing whether similar functionally relevant introgression occurred in North African populations will require targeted, haplotype‐level analyses, which are beyond the scope of this study and cannot be robustly addressed with our current low‐coverage dataset. A key open question, is therefore whether introgression from *M. spretus* contributed adaptive genetic variants that facilitated the ecological success or niche expansion of the house mouse in Africa, an avenue that future studies should explore to fully understand the role of hybridisation in this commensal mammal invasion.

The complex colonisation history revealed here mirrors patterns observed in human genomic studies across Africa, which document layers of ancient and recent admixture associated with ‘back‐to‐Africa’ movements, Arab expansion, trans‐Saharan trade, Morisco expulsions, and European colonial gene flow (Henn et al. 2012; Harich et al. 2010; Oteo‐Garcia et al. 2025; Vicente et al. 2019). Similar human‐mediated structuring has shaped house mouse populations globally, from New Zealand and eastern North America to remote oceanic islands (Gray et al. 2014; Hardouin et al. 2010; Veale et al. 2018; Agwamba and Nachman 2023). Comparable genomic imprints of human dispersal are also evident in other commensal species, including *Rattus rattus* , *Rattus exulans* , and the domestic cat (Matisoo‐Smith et al. 1998; Brouat et al. 2014; Ottoni et al. 2017). Together, these convergent histories highlight how human expansion has not only structured our own demography but has also left lasting genomic signatures in the commensal species that have accompanied us throughout history. This reinforces the value of commensal mammals as biological archives of human‐mediated dispersal and as models for understanding how invasion histories are shaped by repeated introductions, admixture, and shifting connectivity through time.

Overall, our findings highlight a complex genomic legacy of the house mouse invasion in Africa, shaped by multiple, temporally staggered introductions from genetically differentiated West Asia and European lineages, each carrying distinct genomic backgrounds and varying levels of *M. spretus* ancestry. Human‐facilitated dispersal along maritime and trans‐Saharan routes further contributed to admixture and population structure. In North Africa, secondary contact with native *M. spretus* likely added another layer of gene flow, while local demographic histories, reflected in patterns of heterozygosity and inbreeding, have shaped genomic variation inland. By integrating genome‐wide data with demographic reconstructions and introgression analyses, our study sheds light on the evolutionary processes underlying a successful commensal invasion and highlights how human movement can generate complex genomic mosaics in invasive species.

## Author Contributions

D.P.M.: methodology, analysis, writing – original draft; P.G.: methodology, analysis; M.J.M.L.: analysis; F.D.: sampling; G.D.: sampling, review and editing; J.P.Q.: sampling; A.D.: sampling; Y.N.: sampling; M.K.: sampling; C.M.B.: sampling, J.M.: sampling, S.A.A.: sampling; S.B.: sampling; M.G.: sampling; P.C.: sampling; B.N.: sampling; C.M.S.: sampling, review and editing; L.B.: sampling; L.G.: sampling, review and editing; V.R.: writing – original draft, conceptualisation, supervision; F.P.: sampling, writing‐original draft, conceptualisation, supervision; C.B.: sampling, writing – original draft, conceptualisation and supervision. All authors proofread and approved the final version of the manuscript.

## Funding

This work was supported by Agence Nationale de la Recherche, Institut de Recherche pour le Développement, Institut National de Recherche pour l'Agriculture, l'Alimentation et l'Environnement.

## Ethics Statement

Sampling and animal procedures were conducted in accordance with French regulations and the regulations in force in each country where the study was carried out. All procedures were performed under the institutional authorisation for the use of animals for scientific purposes held by the Centre de Biologie pour la Gestion des Populations (CBGP; authorisation n°E34‐169‐001). Permission to enter and work within villages was obtained from the appropriate institutional, traditional, and/or familial authorities. Some samples included in this study were collected prior to the implementation of the Nagoya Protocol or prior to the implementation of applicable national access and benefit‐sharing (ABS) regulations and were therefore considered outside the scope of these requirements. For samples potentially falling within the scope of the Nagoya Protocol, the necessary due diligence procedures were undertaken with the relevant national authorities. All animal‐related procedures were carried out following the official ethical guidelines of the American Society of Mammalogists. Additional details on sampling origin, collection dates, and processing are provided in Note S1 and Table S1.

## Conflicts of Interest

The authors declare no conflicts of interest.

## Supporting information


**Figure S1:** Flowchart summarizing dataset construction and analytical framework used in this study. The diagram illustrates the two main methodological stages of the study. The first workflow (top panel) outlines the process of sample integration, and the generation of four genomic datasets from 380 whole‐genome sequences, including both newly sequenced and published genomes. The second workflow (bottom panel) details the downstream analyses conducted on each dataset. Software and number of SNPs are specified for each analytical module. Together, the two flowcharts provide a comprehensive overview of the methodological framework employed in this study.
**Figure S2:** Genotype‐likelihood–based phylogeny of *Mus musculus* species complex showing full bootstrap support. Pairwise genetic distances were computed from genotype likelihoods (ANGSD Beagle format) using ngsDist v1.0.10 (Vieira et al. 2016) with 100 bootstrap replicates (block size = 1000 sites). Phylogenetic trees were reconstructed with FastME v2.1.6.1 (Lefort et al. 2015) using the BIONJ algorithm and nearest‐neighbour interchanges (NNI) for topology optimisation. All *M. m. domesticus* samples clustered within a single, well‐supported clade (bootstrap = 100) encompassing three main invasive groups (Northern European, Mediterranean, and Iberian) together with Western Asian populations. The subspecies *M. m. musculus* and *M. m. castaneus* each formed strongly supported clades, and *M. spretus* was recovered as the outgroup.
**Figure S3:** Principal Component Analysis (PCA) using the western house mouse dataset. PCA shows the first three axes of genetic variation based on 317 unrelated genomes, and 19,542 unlinked SNPs. Three main clusters were identified (highlighted with dashed lines): a Northern European cluster, a Mediterranean cluster, and an Iberian cluster, each including their corresponding derived African mouse populations.
**Figure S4:** Best log‐likelihood estimates and admixture proportions estimated using NGSadmix on the western house mouse dataset. The top panel displays the mean log‐likelihood across replicates for each tested value of *K* (*K* = 2 to *K* = 9). Log‐likelihoods do not show a clear plateau or obvious optimum value of *K*, making it difficult to choose a single ‘best’ number of clusters. Accordingly, we present the results for *K* = 2 to *K* = 9 to fully illustrate ancestry structure under different *K* assumptions. The bottom panel displays the results of admixture analyses assuming 2 to 9 ancestral populations based on 317 unrelated genomes and 19,542 unlinked SNPs. At *K* = 2, two major groups emerge: one comprising Iberian populations and another including Mediterranean and Northern European populations. At *K* = 3, Northern European samples separate from the Mediterranean cluster. At *K* = 4, the Niger population from Africa forms its own distinct ancestry. At *K* = 5, African populations from Gabon separate as an additional ancestral cluster. At *K* = 6, populations from West Asia differentiate from the Mediterranean group. At *K* = 7, populations from the United States, Germany, and England separate from other Northern European populations. At *K* = 8, the population from northern France (FRAT) differentiates from the remaining Northern European group. At *K* = 9, Spain (Iberia; SPAC) forms an additional distinct ancestry component.
**Figure S5:** Cross‐entropy estimations and admixture proportions estimated using sNMF on the merged house mouse dataset. The top panel shows the cross‐entropy estimates plotted as a function of the number of assumed ancestral populations (*K*). Admixture proportions for *K* = 2 to *K* = 9 are shown in the bottom panel. At *K* = 2, two major ancestry groups emerge, corresponding to Iberian ancestry versus a combined Mediterranean/Northern European ancestry. At *K* = 3, Mediterranean and Northern European individuals form two distinct ancestry components. At *K* = 4, a West Asian ancestry component differentiates from the Mediterranean group. At *K* = 5, the Niger population from Africa appears as a separate ancestry. At *K* = 6, populations from Gabon further resolve into an additional ancestry cluster. At *K* = 7, Northern European populations become distinct from the derived African ones. At *K* = 8, the population from northern France (FRAT) separates from the remaining Northern European populations. At *K* = 9, the Benin population forms an additional distinct ancestry.
**Figure S6:** Population tree inferred using TreeMix. (A) Second‐order rate of change in log‐likelihood (Δ*m*) estimated with the Evanno method (OptM) across migration edges (*m* = 0–10), based on 25 TreeMix replicates per m. The Δ*m* peak at *m* = 1 supports one migration edge as the optimal model. (B–E) TreeMix trees for *m* = 0 (B, no migration edges), *m* = 1 (C), *m* = 2 (D), and *m* = 3 (E). Inferred migration events are indicated by arrows; arrow direction reflects the inferred direction of gene flow and arrow colour indicates migration weight. Branch lengths represent the amount of genetic drift from the most recent common ancestor. The strongest migration edge is inferred between the Mediterranean and Northern European clusters (C). Additional edges suggest gene flow between the outgroup *M. spretus* and the African population from Mali at *m* = 2 (D) and between *M. spretus* and the African population from Benin at *m* = 3 (E).
**Figure S7:** Population graphs using AdmixtureBayes for house mouse populations. (A), Consensus tree generated by combining nodes with a posterior probability > 90% of appearing in the admixture graph. Square nodes represent admixture events. The percentages in the nodes are the posterior probability that the true graph has a node with the same descendants. (B), Trace plots of the posterior probability, total branch length, and number of admixture events for each chain. (C), The plot of the Gelman‐Rubin convergence diagnostics for three summary statistics after a burn‐in fraction of 50%.
**Figure S8:** *Z*‐scores from *D*‐statistics for introgression. *Z*‐scores greater than 3 indicate significant evidence of gene flow. Values are shown for tests assessing excess allele sharing between African (top panel) and European (bottom panel) *M. m. domesticus* populations (P_2_) and *M. spretus* (P₃), relative to the Iranian M. m. domesticus population (P₁; IRAA), with *M. caroli* as the outgroup. The test was formulated as *D* (P₁, P_2_, P₃, outgroup), where significantly positive values indicate excess allele sharing (i.e., gene flow) between P_2_ and P₃ compared to P₁. Analyses were conducted genome‐wide using all variable sites. Population codes correspond to those shown in Figure 1a.
**Figure S9:** CCR‐based separation profiles for selected population pairs with limited temporal resolution. Cross‐coalescence rates were inferred from Relate genealogies and modelled under the MSMC‐IM framework. For geographically close or recently connected population pairs (e.g., Senegal vs. Mali), the CCR did not decline to 0.5 or yielded unexpectedly deep separation signals. These results indicate limited resolution for very recent divergence events.
**Figure S10:** Inferred effective population size (*Nₑ*) under alternative mutation rates. *Nₑ* trajectories over time were estimated from 357 imputed *M. m. domesticus* genomes using Relate and genome‐wide genealogies. Panels show demographic histories assuming different mutation rates per site per generation: (A) 4.1 × 10^−9^ (Geraldes et al. 2011), and (B) 5.7 × 10^−9^ (Milholland et al. 2017). Varying the mutation rate uniformly rescales both time and *Nₑ* estimates without altering the overall shape or relative ordering of demographic trajectories. Notably, estimates under *μ* = 4.1 × 10^−9^ (panel A) are approximately 1.46 × older than those in panel B, reflecting the proportional rescaling effect of the lower mutation rate. The upper *x*‐axis shows time assuming one generation per year, whereas the lower *x*‐axis shows time assuming two generations per year, illustrating the sensitivity of inferred demographic histories to assumptions about generation time. A word of caution is warranted before drawing definitive historical conclusions. Demographic inference is inherently sensitive to assumptions about mutation rate, generation time, population structure, and gene flow.
**Figure S11:** Demographic histories of Europeans populations. *Nₑ* trajectories over time were inferred from a genome‐wide SFS using Stairway Plot 2. Solid lines represent median *Ne* estimates. A mutation rate of 4.1 × 10^−9^ per site per generation and a two generation per year were used. The upper *x*‐axis shows time assuming one generation per year, whereas the lower *x*‐axis shows time assuming two generations per year.
**Figure S12:** Genome‐wide genetic diversity and Tajima's *D* across populations of *M. m. domesticus*. Genome‐wide genetic diversity was quantified using individual heterozygosity, population‐level nucleotide diversity (*π*), and Watterson's *θ*, all estimated across the autosomes. Heterozygosity is shown for individual mice, with boxplots summarizing population‐level distributions, whereas *π* and *θ* are shown as population‐level estimates. Tajima's *D* was summarised across autosomes and is shown as chromosome‐level distributions. Results are stratified by invasion groups (Iberia, Mediterranean, and Northern Europe) as well as by populations from the native range in West Asia. Within each panel, European populations are denoted by an asterisk (*) in the population label.
**Note S1**. Additional details of sampling collection and processing.
**Note S2**. Additional details on genotype calling and relatedness analysis.
**Note S3**. Genotype imputation and accuracy assessment.

**Table S1:** Genomes of the western house mouse and related subspecies used in this study. The first 303 samples correspond to those new genomes sequenced in this study. Samples were obtained from various scientific collections, including MIVEGEC (Maladies Infectieuses et Vecteurs: Écologie, Génétique, Évolution et Contrôle), ISEM (Institut des Sciences de l'Évolution de Montpellier), and CBGP (Centre de Biologie pour la Gestion des Populations); CBGP‐Small mammal Collection (https://doi.org/10.15454/WWNUPO), and associated information is reported in the related database (see https://bpm‐cbgp.science/). Additional columns indicate whether each sample was included (1) in the respective dataset used for analysis.
**Table S2:** Summary of methods, datasets, and samples used in this study. This table provides an overview of the datasets, sample sizes, and analytical approaches employed in this study. For each analysis, we specify the dataset included, the number of samples, and a brief description of the downstream analyses, indicating whether they were based on genotype likelihoods or genotype calls.
**Table S3:** Results of *D*‐statistics for testing introgression between *Mus spretus* and *M. m. domesticus*. This table presents the results of *D*‐statistics analyses performed to investigate signatures of introgression between house mouse (*M. m. domesticus*) populations and the native *M. spretus* . We used the doAbbababa2 module in ANGSD to compute *D*‐statistics for population triplets (P1, P2, P3), where: P1 = Distant *M. m. domesticus* population (IRAA, Iran); P2 = House mouse populations from Africa and Europe; P3 = *Mus spretus* ; P4 = *Mus caroli* (outgroup). The analysis was conducted genome‐wide using all available variable sites to maximise statistical power. A block jackknife procedure was applied to estimate standard errors and calculate *Z*‐scores. To complement *D*‐statistics and quantify the relative magnitude of introgression, we estimated the admixture proportion *f* (Martin et al. 2015), which scales the observed excess of ABBA over BABA sites relative to the expectation under complete admixture.
**Table S4:** List of 114 *Mus musculus domesticus* samples genotyped using SNP arrays and included in the merged dataset. Genotype data for these European individuals were obtained from: (Morgan et al. 2022). Sample names correspond to those provided in the Supporting Information of the original publication. Additional details about the selected samples can be found in the original publication, its Supporting Information, and the associated online repositories.

## Data Availability

All data used in this study are described in the main Article and Supporting Information. Samples locations and their sources are described in Table S1. Raw FASTQ files generated in this study, along with associated metadata, are publicly available in the European Nucleotide Archive (ENA) under project accession number PRJEB90815. Additional datasets include raw sequencing data from *M. m. domesticus* individuals from Europe (PRJEB9450) (Harr et al. 2016) and the United States (PRJNA397406) (Phifer‐Rixey et al. 2018); *M. m. castaneus* from India (PRJEB2176) (Harr et al. 2016); *M. m. musculus* from Kazakhstan (PRJEB11742) (Harr et al. 2016); *M. spretus* from Spain (PRJEB11742) (Harr et al. 2016); and *M. caroli* reads (PRJEB14895) (Thybert et al. 2018). Genotype data from 114 *M. m. domesticus* individuals from Europe (Morgan et al. 2022) are available from the Dryad Repository (https://doi.org/10.5061/dryad.ncjsxkswt). The chromosome‐level reference genome assembly of the house mouse (GRCm39/mm39) used in this study is available in the NCBI database under accession number GCF_000001635.27. Code used for analyses within this study are publicly available on Github: https://github.com/danielpovedam/AfricaMicetral. A description of software and their version numbers can be found in Methods and Table S2. All relevant genomic datasets are accessible via Figshare repository https://doi.org/10.6084/m9.figshare.29485058.
