# Supplementary material for: Multiple Eurasian Origins and Admixture Shaped the Genomic Legacy of the House Mouse Invasion in Africa
Source: Mol Ecol. 2026 Aug 13;35(16):e70507. doi: 10.1111/mec.70507 (PMC13473817; doi:10.1111/mec.70507)
Supplement: Supplementary file 1 [file MEC-35-e70507-s002.docx]

Supplementary information for

**Multiple Eurasian origins and admixture shaped the genomic legacy of the house mouse invasion in Africa**

Daniel Poveda-Martinez^1*^, Philippe Gauthier^1^, Margaux J. M. Lefebvre^2^, Fanny Degrugillier^3^, Gauthier Dobigny^1,4^, Jean-Pierre Quéré^1^, Ambroise Dalecky^5,6^, Youssoupha Niang^1,5^, Mamadou Kane^1,5^, Clark Mbou-Boutambe^7^, Joa Mangombi^7^, Solimane Ag Atteynine^8^, Sylvestre Badou^9^, Madougou Garba^10^, Pierre Caminade^11^, Barthélémy Ngoubangoye^7^, Carole M. Smadja^11^, Larson Boundenga^7^, Laurent Granjon^1^, Virginie Rougeron^12,13^, Franck Prugnolle^12,13*^, Carine Brouat^1*^

^1^CBGP, Univ Montpellier, IRD, CIRAD, INRAE, Institut Agro, Montpellier, France.

^2^Department of Archaeogenetics, Max Planck Institute for Evolutionary Anthropology, Leipzig, Germany.

^3^Laboratoire MIVEGEC, Univ Montpellier, CNRS-UM, IRD, Montpellier, France

^4^Institut Pasteur de Madagascar, Unité Peste, Antananarivo, Madagascar.

^5^BIOPASS 2, IRD, Cirad, ISRA, Université Gaston Berger, Saint-Louis, Sénégal.

^6^LPED, Aix Marseille Université, IRD, Marseille, France.

^7^Centre Interdisciplinaires de Recherches Médicales de Franceville (CIRMF), Franceville, Gabon.

^8^Institut d’Economie Rurale (IER), Sotuba, Mali.

^9^Ecole Polytechnique d’Abomey Calavi (EPAC), Université d’Abomey Calavi, d'Abomey-Calavi, Bénin.

^10^Direction Générale de la Protection des Végétaux (DGPV), Niamey, Niger.

^11^Institut des Sciences de l'Evolution de Montpellier (ISEM), Univ Montpellier, IRD, Montpellier, France.

^12^REHABS, International Research Laboratory, CNRS-NMU, George Campus, Nelson Mandela University, George, South Africa.

^13^Sustainability Research Unit, George Campus, Nelson Mandela University, George, South Africa

*Corresponding authors: Daniel Poveda-Martinez, [danielpovedam@gmail.com](mailto:danielpovedam@gmail.com); Franck Prugnolle, [franck.prugnolle@cnrs.fr](mailto:franck.prugnolle@cnrs.fr); Carine Brouat, [carine.brouat@ird.fr](mailto:carine.brouat@ird.fr).

**This PDF file includes:**

Figs. S1 to S12.

Supplementary Note S1 to S3.

Legends for supplementary tables S1 - S4.

**Other Supplementary Materials for this manuscript include the following:**

Tables S1 - S4

**Fig. S1. Flowchart summarizing dataset construction and analytical framework used in this study**. The diagram illustrates the two main methodological stages of the study. The first workflow (top panel) outlines the process of sample integration, and the generation of four genomic datasets from 380 whole-genome sequences, including both newly sequenced and published genomes. The second workflow (bottom panel) details the downstream analyses conducted on each dataset. Software and number of SNPs are specified for each analytical module. Together, the two flowcharts provide a comprehensive overview of the methodological framework employed in this study.

**Fig. S2. Genotype-likelihood–based phylogeny of *Mus musculus* species complex showing full bootstrap support.** Pairwise genetic distances were computed from genotype likelihoods (ANGSD Beagle format) using ngsDist v1.0.10 (Vieira et al. 2016) with 100 bootstrap replicates (block size = 1,000 sites). Phylogenetic trees were reconstructed with FastME v2.1.6.1 [(Lefort et al. 2015)](https://paperpile.com/c/n5Q96V/biIC) using the BIONJ algorithm and nearest-neighbor interchanges (NNI) for topology optimization. All *M. m. domesticus* samples clustered within a single, well-supported clade (bootstrap = 100) encompassing three main invasive groups (Northern European, Mediterranean, and Iberian) together with Western Asian populations. The subspecies *M. m. musculus* and *M. m. castaneus* each formed strongly supported clades, and *M. spretus* was recovered as the outgroup.

**Fig. S3. Principal Component Analysis (PCA) using the western house mouse dataset.** PCA shows the first three axes of genetic variation based on 317 unrelated genomes, and 19,542 unlinked SNPs. Three main clusters were identified (highlighted with dashed lines): a Northern European cluster, a Mediterranean cluster, and an Iberian cluster, each including their corresponding derived African mouse populations

**Fig. S4. Best log-likelihood estimates and admixture proportions estimated using NGSadmix on the western house mouse dataset**. The top panel displays the mean log-likelihood across replicates for each tested value of K (K = 2 to K = 9). Log-likelihoods do not show a clear plateau or obvious optimum value of K, making it difficult to choose a single “best” number of clusters. Accordingly, we present the results for K = 2 to K = 9 to fully illustrate ancestry structure under different K assumptions. The bottom panel displays the results of admixture analyses assuming 2 to 9 ancestral populations based on 317 unrelated genomes and 19,542 unlinked SNPs. At K=2, two major groups emerge: one comprising Iberian populations and another including Mediterranean and Northern European populations. At K=3, Northern European samples separate from the Mediterranean cluster. At K=4, the Niger population from Africa forms its own distinct ancestry. At K=5, African populations from Gabon separate as an additional ancestral cluster. At K=6, populations from West Asia differentiate from the Mediterranean group. At K=7, populations from the United States, Germany, and England separate from other Northern European populations. At K=8, the population from northern France (FRAT) differentiates from the remaining Northern European group. At K=9, Spain (Iberia; SPAC) forms an additional distinct ancestry component.

**Fig. S5. Cross-entropy estimations and admixture proportions estimated using sNMF on the merged house mouse dataset**. The top panel shows the cross-entropy estimates plotted as a function of the number of assumed ancestral populations (K). Admixture proportions for K=2 to K=9 are shown in the bottom panel. At K=2, two major ancestry groups emerge, corresponding to Iberian ancestry versus a combined Mediterranean/Northern European ancestry. At K=3, Mediterranean and Northern European individuals form two distinct ancestry components. At K=4, a West Asian ancestry component differentiates from the Mediterranean group. At K=5, the Niger population from Africa appears as a separate ancestry. At K=6, populations from Gabon further resolve into an additional ancestry cluster. At K=7, Northern European populations become distinct from the derived African ones. At K=8, the population from northern France (FRAT) separates from the remaining Northern European populations. At K=9, the Benin population forms an additional distinct ancestry.

**Fig. S6. Population tree inferred using TreeMix.** **(A)** Second‐order rate of change in log‐likelihood (Δm) estimated with the Evanno method (OptM) across migration edges (m = 0–10), based on 25 TreeMix replicates per m. The Δm peak at m = 1 supports one migration edge as the optimal model. **(B–E)** TreeMix trees for m = 0 (B, no migration edges), m = 1 **(C)**, m = 2 **(D)**, and m = 3 **(E)**. Inferred migration events are indicated by arrows; arrow direction reflects the inferred direction of gene flow and arrow color indicates migration weight. Branch lengths represent the amount of genetic drift from the most recent common ancestor. The strongest migration edge is inferred between the Mediterranean and Northern European clusters **(C)**. Additional edges suggest gene flow between the outgroup *M. spretus* and the African population from Mali at m = 2 **(D)** and between *M. spretus* and the African population from Benin at m = 3 **(E)**.

**Fig. S7. Population graphs using AdmixtureBayes for house mouse populations**. **(A)**, Consensus tree generated by combining nodes with a posterior probability >90% of appearing in the admixture graph. Square nodes represent admixture events. The percentages in the nodes are the posterior probability that the true graph has a node with the same descendants. **(B)**, Trace plots of the posterior probability, total branch length, and number of admixture events for each chain. **(C)**, The plot of the Gelman-Rubin convergence diagnostics for three summary statistics after a burn-in fraction of 50%.

**Fig. S8. Z-scores from D-statistics for introgression.** Z-scores greater than 3 indicate significant evidence of gene flow. Values are shown for tests assessing excess allele sharing between African (top panel) and European (bottom panel) *M. m. domesticus* populations (P₂) and *M. spretus* (P₃), relative to the Iranian M. m. domesticus population (P₁; IRAA), with *M. caroli* as the outgroup. The test was formulated as D (P₁, P₂, P₃, outgroup), where significantly positive values indicate excess allele sharing (i.e., gene flow) between P₂ and P₃ compared to P₁. Analyses were conducted genome-wide using all variable sites. Population codes correspond to those shown in Fig. 1a.

**Figure S9. CCR-based separation profiles for selected population pairs with limited temporal resolution.** Cross-coalescence rates were inferred from Relate genealogies and modeled under the MSMC-IM framework. For geographically close or recently connected population pairs (e.g., Senegal vs. Mali), the CCR did not decline to 0.5 or yielded unexpectedly deep separation signals. These results indicate limited resolution for very recent divergence events.

**Fig. S10. Inferred effective population size (Nₑ) under alternative mutation rates**. *Nₑ* trajectories over time were estimated from 357 imputed *M. m. domesticus* genomes using Relate and genome-wide genealogies. Panels show demographic histories assuming different mutation rates per site per generation: A) 4.1 × 10⁻⁹ (Geraldes et al., 2011), and B) 5.7 × 10⁻⁹ (Milholland et al., 2017). Varying the mutation rate uniformly rescales both time and *Nₑ* estimates without altering the overall shape or relative ordering of demographic trajectories. Notably, estimates under μ = 4.1 × 10⁻⁹ (panel A) are approximately 1.46 × older than those in panel B, reflecting the proportional rescaling effect of the lower mutation rate. The upper x-axis shows time assuming one generation per year, whereas the lower x-axis shows time assuming two generations per year, illustrating the sensitivity of inferred demographic histories to assumptions about generation time. A word of caution is warranted before drawing definitive historical conclusions. Demographic inference is inherently sensitive to assumptions about mutation rate, generation time, population structure, and gene flow.

**Fig. S11. Demographic histories of Europeans populations.** *Nₑ* trajectories over time were inferred from a genome-wide SFS using Stairway Plot 2. Solid lines represent median *Ne* estimates. A mutation rate of 4.1 × 10⁻⁹ per site per generation and a two generation per year were used. The upper x-axis shows time assuming one generation per year, whereas the lower x-axis shows time assuming two generations per year.

**Fig. S12. Genome-wide genetic diversity and Tajima’s D across populations of *M. m. domesticus*.** Genome-wide genetic diversity was quantified using individual heterozygosity, population-level nucleotide diversity (π), and Watterson’s θ, all estimated across the autosomes. Heterozygosity is shown for individual mice, with boxplots summarizing population-level distributions, whereas π and θ are shown as population-level estimates. Tajima’s D was summarized across autosomes and is shown as chromosome-level distributions. Results are stratified by invasion groups (Iberia, Mediterranean, and Northern Europe) as well as by populations from the native range in West Asia. Within each panel, European populations are denoted by an asterisk (*) in the population label.

**Supplementary Notes**

**Note S1. Additional details of sampling collection and processing.**

Sampling was conducted in urban and peri-urban environments across multiple African countries, using standardized live-trapping procedures to target commensal Mus musculus domesticus associated with human settlements. Permission to enter and work within villages in Africa was systematically obtained from the appropriate institutional, traditional, and/or familial authorities. Trapping sessions were conducted in accordance with the requirements of both local and French legislation. All protocols used in this study received prior explicit approval from the relevant institutional committee at the Centre de Biologie pour la Gestion des Populations (CBGP; agreement for the use of animals for scientific purposes: E34-169-001). Some samples included in this study were collected prior to the implementation of the Nagoya Protocol or prior to the implementation of applicable national access and benefit-sharing (ABS) regulations and were therefore considered outside the scope of these requirements. For samples potentially falling within the scope of the Nagoya Protocol, the necessary due diligence procedures were undertaken with the relevant national authorities. In Mali, the project including these samples was approved by the ethics committee of the National Institute of Public Health of Mali (decision n°18/2021/CE-INSP). Prior informed consent was also obtained, in accordance with the provisions of the Nagoya Protocol signed by Mali, from the National Director of Water and Forests of Mali (letter 0976/MEADD-DNEF, 25 November 2021). All animal-related procedures were carried out following the official ethical guidelines of the American Society of Mammalogists. Additional details on sample collections are provided in Table S1.

Genomic DNA was extracted from a mixture of organs (kidney, spleen, and paw) using the Qiagen DNeasy Blood & Tissue Kit, following the manufacturer’s instructions (Qiagen, Inc., Valencia, CA, USA). DNA concentration was quantified using a Qubit 2.0 Fluorometer (Life Technologies). Whole-genome sequencing was performed to all samples at low coverage (lcWGS) (2,4× mean depth, individual sample depths are provided in Table S1) using 150 bp paired-end reads mode on the Illumina NovaSeq 6000 platform (Illumina Inc., San Diego, CA, USA) at the MGX-Montpellier, France, GenomiX Core Facility. Whole-genome sequencing data were assessed using FastQC v0.11.9 and MultiQC v1.13 to evaluate the quality of paired-end raw reads and potential adaptor contamination before and after data processing.

**Note S2. Additional details on genotype calling and relatedness analysis**

We used the Snakemake pipeline loco-pipe (Zhou et al. 2024) to standardize and automate genotype-likelihood estimation in ANGSD across all samples. Loco-pipe implements recommended best practices for low-coverage whole-genome sequencing data and ensures that identical filtering and file-generation steps are applied across individuals, thereby minimizing potential batch effects (Lou et al. 2021; Zhou et al. 2024). Genotype likelihoods were estimated using the GATK model implemented in ANGSD (-GL 2). Missing data in the ANGSD derived datasets were handled through site-level sample-representation and read-depth filters. In the snp_calling_global rule of loco-pipe, we required at least 50% of individuals in the dataset to have a read depth of at least one read at a site for that site to be retained (minind_proportion = 0.5; mindepthind = 1). Sites represented in fewer than half of the individuals were therefore excluded from the genotype-likelihood datasets. This filter provided the main control for missing data in the ANGSD-based analyses. As a first step, loco-pipe computes site-wise sequencing depth across the full dataset using ANGSD. Only sites falling within the retained depth thresholds for low- and medium/high-depth samples were kept for downstream analyses. Using the snp_calling_global rule, we estimated allele frequencies and genotype likelihoods across the autosomes using the following ANGSD flags: -minMapQ 30, -minQ 30, -remove_bads 1, -uniqueOnly 1, -only_proper_pairs 1, -GL 2, -doMaf 1, -doMajorMinor 1, and -doGlf 2. SNPs were retained using a minimum minor allele frequency of 0.05 (minmaf = 0.05) and a SNP discovery threshold of P < 1 × 10⁻⁶ (-SNP_pval 1e-6). Additionally, a position file of all retained SNPs (only variable sites) was created from the minor allele frequencies output to be used in downstream analysis (hereafter, FullSNPs). To reduce linkage disequilibrium, we ran the subset_snp_list_global rule from loco-pipe to thin the SNP list and beagle-formatted genotype likelihood files picking the first SNP out of the 1,000 continuous SNPs (SubsetSNPs), for analyses requiring unlinked sites certain downstream analyses that include all samples (Zhou et al. 2024).

In addition to filtering on depth and quality, we considered relatedness between samples, where we identified and removed potential relatives and duplicated samples using the methodology described in ngsRelate v2 (Hanghøj et al. 2019). First, we estimated the genotype likelihoods in ANGSD for the reduced set of SNPs for all samples (-sites = SubsetSNPs) in format BCF (-dobcf 1), using the flags: -skipTriallelic 1 -minmaf 0.05 -doCounts 1 -GL 2 -doGlf 3 -doMajorMinor 1 -doMaf 1 -SNP_pval 1e-6 -dobcf 1 -doPost 1. NgsRelate was used to estimate allele frequencies from genotype likelihood using the PL (Phred-scaled likelihoods for genotypes) tag, and calculate for each pair of samples the R0, R1 and KING-robust kinship (Waples et al. 2019), to identify close relatives. Sample pairs with a KING-robust kinship coefficient > 0.177 were considered first-degree relatives, such as parent–offspring pairs or full siblings, and were excluded to avoid bias in downstream analyses. In such cases, one individual per pair was randomly retained to preserve a set of unrelated samples. This filtering step resulted in the removal of 40 samples, which are indicated in Table S1. After relatedness filtering, the final multispecies dataset comprised 340 unrelated whole genomes spanning *M. musculus* subspecies and *M. spretus* across 34 populations, with an average of 10.0 individuals per population. This dataset contained 23.1 million SNPs, including 23,110 unlinked SNPs. The western house mouse dataset comprised 317 unrelated whole genomes of the focal subspecies *M. m. domesticus* across 31 populations, with an average of 10.2 individuals per population. This dataset contained 19.5 million SNPs, including 19,542 unlinked SNPs.

**Note S3. Genotype imputation and accuracy assessment**

We performed genotype imputation with STITCH (Sequencing To Imputation Through Constructing Haplotypes) (Davies et al. 2016) to obtain high-confidence genotype calls from our predominantly low-coverage whole-genome sequencing (lcWGS) data. STITCH infers haplotypes and genotypes directly from sequencing reads using an expectation–maximization procedure within a hidden Markov model framework, and therefore does not require an external reference haplotype panel. Imputation was necessary because several downstream analyses (e.g., genealogy-based demographic inference with Relate; Speidel et al. 2019) require called genotypes. Imputation was applied to ~357 *M. m. domesticus* genomes (newly sequenced lcWGS, mean depth ~2.4×, together with published genomes spanning ~5×–21×). We restricted imputation to high-quality variable sites previously identified during SNP discovery in ANGSD: only sites passing initial QC and detected as polymorphic under a stringent SNP-calling threshold (p < 1×10⁻⁶, Full_SITES_) were considered. This strategy reduces the impact of spurious variants driven by stochastic sampling at low coverage and ensures that imputation is focused on a curated set of informative sites. We retained only imputed genotypes with an imputation score >0.9, generating the imputed house mouse dataset used in analyses requiring discrete genotypes. After imputation, the dataset contained 19,998,106 SNPs genome-wide. To retain high-confidence imputed variants, we filtered SNPs using an imputation information score ≥ 0.9. We further limited missing data by retaining only sites genotyped in at least 90% of individuals. After these filtering steps, the final imputed house mouse dataset contained 14,981,900 SNPs, corresponding to ~74.9% of the initial imputed SNP set. We subsequently assessed individual-level missingness using VCFtools (--missing-indv). Across individuals, missingness ranged from 0.0055 to 0.1327, with a mean of 0.0254, indicating that missing genotype calls were generally low after site-level filtering. This filtered imputed dataset was used for analyses requiring discrete genotypes.

To evaluate imputation accuracy, we used 19 high-coverage genomes (mean depth ~21×) from three wild European populations of *M. m. domesticus* (Harr et al. 2016; sample details in Table S1). For these individuals, high-coverage reads were processed with fastp to remove adapters and low-quality bases, aligned to GRCm39 using bwa-mem2, and genotypes were called with GATK v4.2.6.1 (McKenna et al. 2010). These high-confidence calls constituted the validation dataset against which imputed genotypes were benchmarked. To assess accuracy under conditions matching our lcWGS data, we downsampled the 19 high-coverage BAM files to ~2.4× mean depth using SAMtools v1.20. These downsampled genomes were then included in the STITCH imputation jointly with the full dataset, ensuring that imputation performance was evaluated in the same analytical context (site set, LD structure, sample composition) as the study-wide imputation. We compared imputed genotypes from the downsampled data to the corresponding high-coverage GATK genotypes on a per-chromosome basis. Shared SNPs between the imputed and validation datasets were identified using bcftools isec v1.9, yielding ~13.7 million intersected SNPs. We quantified imputation performance using two complementary measures (Browning & Browning 2009): Dosage correlation (R²): squared Pearson correlation between imputed genotype dosages and true genotypes across individuals. Genotype concordance (GC): proportion of genotypes that matched exactly between the imputed and validation datasets. Across chromosomes, STITCH performed consistently well, with mean R² = 0.834 and GC = 0.947, indicating robust recovery of true genotypes from lcWGS data under our filtering strategy and confirming suitability of the imputed dataset for downstream analyses requiring called genotypes.

**Imputation accuracy per chromosome.** Mean genotype concordance (GC) per chromosome, defined as the proportion of exact matches between imputed and true genotypes across individuals (top panel). Mean imputation accuracy (R²) per chromosome, calculated as the squared Pearson correlation between true genotypes and imputed dosages across 19 validation individuals (bottom panel). Error bars represent inter-individual variability.

**Additional supplementary material includes:**

**Table S1. Genomes of the western house mouse and related subspecies used in this study**. The first 303 samples correspond to those new genomes sequenced in this study. Samples were obtained from various scientific collections, including MIVEGEC (Maladies Infectieuses et Vecteurs: Écologie, Génétique, Évolution et Contrôle), ISEM (Institut des Sciences de l'Évolution de Montpellier), and CBGP (Centre de Biologie pour la Gestion des Populations; CBGP-Small mammal Collection (https://doi.org/10.15454/WWNUPO), and associated information is reported in the related database (see https://bpm-cbgp.science/). Additional columns indicate whether each sample was included (1) in the respective dataset used for analysis.

**Table S2. Summary of methods, datasets, and samples used in this study.** This table provides an overview of the datasets, sample sizes, and analytical approaches employed in this study. For each analysis, we specify the dataset included, the number of samples, and a brief description of the downstream analyses, indicating whether they were based on genotype likelihoods or genotype calls.

**Table S3. Results of *D-Statistics* for testing introgression between *Mus spretus* and *M. m. domesticus***. This table presents the results of *D-statistics* analyses performed to investigate signatures of introgression between house mouse *(M. m. domesticus*) populations and the native *M. spretus*. We used the doAbbababa2 module in ANGSD to compute *D-statistics* for population triplets (P1, P2, P3), where: P1= Distant *M. m. domesticus* population (IRAA, Iran); P2= House mouse populations from Africa and Europe; P3= *Mus spretus* populations (spretusA, *M. spretus* from Africa, Morocco - Agadir); P4= *Mus caroli* (outgroup). The analysis was conducted genome-wide using all available variable sites to maximize statistical power. A block jackknife procedure was applied to estimate standard errors and calculate Z-scores. To complement D-statistics and quantify the relative magnitude of introgression, we estimated the admixture proportion f (Martin et al. 2015), which scales the observed excess of ABBA over BABA sites relative to the expectation under complete admixture.

**Table S4. List of 114 *Mus musculus domesticus* samples genotyped using SNP arrays and included in the merged dataset.** Genotype data for these European individuals were obtained from: [(Morgan et al. 2022)](https://paperpile.com/c/n5Q96V/Fy1QX). Sample names correspond to those provided in the supplementary information of the original publication. Additional details about the selected samples can be found in the original publication, its supplementary materials, and the associated online repositories.

**References**

[Browning, Brian L., and Sharon R. Browning. 2009. “A Unified Approach to Genotype Imputation and Haplotype-Phase Inference for Large Data Sets of Trios and Unrelated Individuals.” *American Journal of Human Genetics* 84 (2): 210–223.](http://paperpile.com/b/n5Q96V/ap53K)

[Davies, Robert W., Jonathan Flint, Simon Myers, and Richard Mott. 2016. “Rapid Genotype Imputation from Sequence without Reference Panels.” *Nature Genetics* 48 (8): 965–969.](http://paperpile.com/b/n5Q96V/4TI9O)

[Druet, T., and M. Gautier. 2017. “A Model-Based Approach to Characterize Individual Inbreeding at Both Global and Local Genomic Scales.” *Molecular Ecology* 26 (20): 5820–5841.](http://paperpile.com/b/n5Q96V/0F0A)

[High-Throughput and Cost-Effective Genotyping by Low-Coverage Whole Genome Sequencing with Genotype Imputation in Pacific Oyster, Crassostrea Gigas. 2024. *Aquaculture* 591 (October): 741134.](http://paperpile.com/b/n5Q96V/CdUK4)

Filipe G. Vieira, Florent Lassalle, Thorfinn S. Korneliussen, Matteo Fumagalli, Improving the estimation of genetic distances from Next-Generation Sequencing data, Biological Journal of the Linnean Society, Volume 117, Issue 1, January 2016, Pages 139–149

[Lefort, Vincent, Richard Desper, and Olivier Gascuel. 2015. “FastME 2.0: A Comprehensive, Accurate, and Fast Distance-Based Phylogeny Inference Program.” *Molecular Biology and Evolution* 32 (10): 2798–2800.](http://paperpile.com/b/n5Q96V/biIC)

Lou, R. N., Jacobs, A., Wilder, A. P., & Therkildsen, N. O. (2021). A beginner's guide to low‐coverage whole genome sequencing for population genomics. Molecular Ecology, 30(23), 5966-5993.

[Meisner, Jonas, Anders Albrechtsen, and Kristian Hanghøj. 2021. “Detecting Selection in Low-Coverage High-Throughput Sequencing Data Using Principal Component Analysis.” *BMC Bioinformatics* 22 (1): 470.](http://paperpile.com/b/n5Q96V/hma8)

[Morgan, Andrew P., Jonathan J. Hughes, John P. Didion, et al. 2022. “Population Structure and Inbreeding in Wild House Mice (*Mus Musculus*) at Different Geographic Scales.” *Heredity* 129 (3): 183–194.](http://paperpile.com/b/n5Q96V/Fy1QX)

[Speidel, Leo, Marie Forest, Sinan Shi, and Simon R. Myers. 2019. “A Method for Genome-Wide Genealogy Estimation for Thousands of Samples.” *Nature Genetics* 51 (9): 1321–1329.](http://paperpile.com/b/n5Q96V/yxPO)

[Teng, Jun, Changheng Zhao, Dan Wang, et al. 2022. “Assessment of the Performance of Different Imputation Methods for Low-Coverage Sequencing in Holstein Cattle.” *Journal of Dairy Science* 105 (4): 3355–3366.](http://paperpile.com/b/n5Q96V/F0gI)

[Wang, Ke, Iain Mathieson, Jared O’Connell, and Stephan Schiffels. 2020. “Tracking Human Population Structure through Time from Whole Genome Sequences.” *PLoS Genetics* 16 (3): e1008552.](http://paperpile.com/b/n5Q96V/PAWo)

Zhou, Z. T., Owens, G. L., Larson, W. A., Lou, R. N., & Sudmant, P. H. (2024). loco-pipe: an automated pipeline for population genomics with low-coverage whole-genome sequencing. Bioinformatics Advances, 4(1), vbae098.
